# Tobacco Smoking and Smoke‐Free Products as Risk Factors for Dental Implants: A Systematic Review

**DOI:** 10.1111/clr.70108

**Published:** 2026-03-03

**Authors:** Calciolari Elena, Corbella Stefano, Dourou Marina, Ercal Pinar, Donos Nikolaos

**Affiliations:** ^1^ Centre for Oral Clinical Research, Institute of Dentistry, Faculty and Medicine and Dentistry Queen Mary University of London London UK; ^2^ Dental School, Department of Medicine and Surgery Università di Parma Parma Italy; ^3^ Department of Biomedical, Surgical and Dental Sciences Università degli Studi di Milano Milano Italy; ^4^ IRCCS Ospedale Galeazzi Sant'Ambrogio Milan Italy

**Keywords:** dental implants, risk factors, smoking, tobacco use, vaping, electronic cigarettes

## Abstract

**Objectives:**

This systematic review aimed to investigate the effect of tobacco smoking and smoke‐free products as risk factors for dental implants.

**Materials and Methods:**

Three databases were searched to identify studies reporting on the risk of implant failure/survival in tobacco smokers or smoke‐free users as compared to non‐smokers in studies with ≥ 1 year of follow‐up post‐implant loading. Data on biological complications and radiographic peri‐implant crestal bone loss (CBL) were also collected. Meta‐analyses computed the Odds Ratio (OR) of implant survival with its confidence interval by applying the DerSimonian and Laird's random effect method, with *p* set at < 0.01.

**Results:**

Forty‐five articles reporting on 44 studies were included, 41 of which reported on the effect of cigarette smoking, with a follow‐up from 1 to up to 17 years. Overall, a significantly reduced implant survival both at implant and patient level was indicated in cigarette smokers (OR = 0.40, 95% CI 0.27; 0.61, *p* < 0.001 and OR = 0.43, 95% CI 0.20; 0.90, *p* = 0.02, respectively). An increased CBL of 0.64 mm (95% CI 0.29; 0.99, *p* < 0.001) was also suggested in cigarette smokers, and the majority of studies reported a higher incidence of peri‐implantitis. Insufficient data are available for smoke‐free users.

**Conclusions:**

Although tobacco smoking is not considered an absolute contraindication for implant therapy, evidence indicates that it has a detrimental effect on peri‐implant tissues, leading to an increased risk of implant failure and crestal bone loss. Individual behavioural counselling for smoking cessation should always be integrated in the treatment plan of patients receiving implant‐supported rehabilitations.

**Trial Registration:**

PROSPERO number: CRD42024628116

## 1 Introduction

Despite clinical data suggest that dental implants are successful options to rehabilitate partial and complete edentulism (Albrektsson et al. 2012), a number of risk factors can affect short‐ and long‐term implant success, including quality and volume of available bone, location, augmentation procedures, as well as local/systemic and environmental conditions, such as history of periodontitis, uncontrolled diabetes mellitus and tobacco smoking (Bornstein et al. 2009; Sousa et al. 2016; Do et al. 2020; Zangrando et al. 2015; Carra et al. 2022; Annunziata et al. 2025). Previous studies have clearly proved the detrimental effects of tobacco smoking on the oral cavity (including periodontal tissues), owing to its effects on the oral microbiota, tissue homeostasis, vascularization and fibroblast function (Apatzidou 2022). Systematic reviews and meta‐analyses have also reported an increased peri‐implant marginal bone loss, a higher risk of dental implant failure and of peri‐implantitis (Naseri et al. 2020; Moraschini and Barboza 2016; Reis et al. 2023; Alfadda 2018; Chrcanovic et al. 2015) in smokers. Remarkably, in the recent review by Carra et al. (2023) on the efficacy of risk factor control for primordial and primary prevention of peri‐implant diseases they could only identify one study that reported on the occurrence of peri‐implant diseases and it reported a lower rate of peri‐implant mucositis (43.9% vs. 48.6%) and of peri‐implantitis (19.7% vs. 30.5%) in former smokers compared to current smokers (Costa et al. 2022). The above mentioned study suggested a direct association between cumulative smoking exposure and risk for peri‐implantitis, with a significant decrease in peri‐implantitis risk as the years of smoking cessation increased. Despite the paucity of evidence currently available on the benefit of smoking cessation on the prevention of peri‐implant diseases, interventions promoting smoking cessation in dental practices are highly recommended, also in consideration of the several harmful consequences of smoking for the overall health of patients (Herrera et al. 2023).

In recent years, there has been an increased use of a large range of smoke‐free products (Bold et al. 2018; Dinardo and Rome 2019), which eliminate the burning of tobacco (and related ashes). They can be broadly distinguished into heated tobacco products, where heated tobacco units generate a nicotine‐containing vapor without burning tobacco, and in e‐vapor products, where a liquid (often containing nicotine but not tobacco) is heated to create a vapor/aerosol that is inhaled. Most of smoke‐free products fit into the inhalable category (e.g., e‐cigarettes, vaping), but not all of them are inhalable (e.g., chewing tobacco, dip, nicotine pouches). Compared to traditional tobacco smoking, the harm associated with electronic nicotine delivery systems may be underestimated due to the reduced ability to control vaping behaviour, ease of access, fewer vaping area restrictions, and better taste (Zhang and Wen 2023).

Habitual use of smokeless tobacco products has been associated with oral inflammatory conditions, such as oral precancer, cancer, and periodontitis (Ramoa et al. 2017). The effect of habitual use of smokeless tobacco products on the success and survival of dental implants remains uncertain (Javed et al. 2019), although few reviews pointed out the likely detrimental effect of such habits on dental implants (Akram et al. 2019; Youssef et al. 2023). Current guideline by the European Federation of Periodontology underlines that there is little evidence to support the contention that using e‐cigarettes or the habit of water pipe smoking is associated with a decreased risk for peri‐implant diseases compared with cigarette smoking (Herrera et al. 2023).

The present systematic review and meta‐analysis aimed to provide updated evidence on the effect of smoking and smoke‐free products on implant survival.

## 2 Material and Methods

The study protocol was registered in PROSPERO before full‐text screening and is in line with the Cochrane Handbook (Higgins, Thomas, et al. 2024).

### 2.1 Focused Questions

The review aimed to answer two focused questions:

- Focused question 1 (FQ1): What is the risk of implant failure in tobacco smokers based on RCTs, CCTs, case–control and cohort studies with at least 1 year of follow‐up post‐implant loading?
- Focused question 2 (FQ2): What is the risk of implant failure in smoke‐free users, including users of heated tobacco products, e‐vapor products and other non‐inhalable products (e.g., tobacco chewing, nicotine pouches, snus) based on RCTs, CCTs, case–control, cross‐sectional and cohort studies with at least 1 year of follow up post implant loading?

### 2.2 Inclusion/Exclusion Criteria

The following inclusion/exclusion criteria (based on the PECOS) were considered for FQ1:

Population: Adult (> 18 years old) patients that received dental implants (single units and multiple units up to full arches) and including also cases where bone regeneration was performed concomitant or before implant placement.

Exposure: Current tobacco smokers (any type of tobacco smoking, including cigarettes, pipes, cigars, bidis, hookah) or smokers that quit during the study.

Comparison: Never smokers or former smokers (quit since at least 1 year). Smoke‐free users were excluded.

Outcome: Primary outcome was implant failure/survival (relative risk, odds ratio, hazard ratio) at least 1 year post‐implant loading; secondary outcomes included: peri‐mucositis and peri‐implantitis risk, radiographic peri‐implant marginal bone loss; other biological complications (e.g., soft tissue dehiscence, early implant failure); changes in peri‐implant crevicular fluid (PICF) markers, changes in microbial plaque composition, patient‐reported outcome measures (PROMs).

Study Design: RCTs, CCTs, case–control (retrospective) studies (minimum of 30 patients—15 test and 15 controls), cross‐sectional studies (minimum of 30 patients—15 test and 15 controls), cohort studies (minimum of 30 patients) with at least 1 year of follow‐up post‐implant loading.

The following inclusion/exclusion criteria (based on the PECOS) were considered for FQ2:

Population: Adult (> 18 years old) patients that received dental implants (single units and multiple units up to full arches) and including also cases where bone regeneration was performed concomitant or before implant placement.

Exposure: Current smoke‐free users, including users of heated tobacco, e‐cigarettes, vapers and non‐inhalable products (e.g., chewing tobacco, dip, dissolvable, snuff, snus, nicotine pouches) or smoke‐free users that quit during the study.

Comparison: Non‐users of smoke‐free products or former users (quit since at least 1 year). Tobacco smokers were not included.

Outcome: Primary outcome was implant failure/survival (relative risk, odds ratio, hazard ratio) at least 1 year post‐implant loading; secondary outcomes included: peri‐mucositis and peri‐implantitis risk, radiographic peri‐implant crestal bone loss (CBL); other biological complications (e.g., soft tissue dehiscence, early implant failure); changes in PICF markers, changes in microbial plaque composition, PROMs.

Study Design: RCTs, CCTs, case–control (retrospective) studies (minimum of 30 patients—15 test and 15 controls), cross‐sectional studies (minimum of 30 patients—15 test and 15 controls), cohort studies (minimum of 30 patients).

Owing to the nature of exposure, it was expected that no RCTs would be identified.

### 2.3 Search Methods for Study Identification

A sensitive strategy was developed aiming to identify all studies meeting the inclusion/exclusion criteria.

The research strategy included terms related to the Population and the Exposure investigated in this review, which were combined with the boolean operator “AND”.

Three main databases were searched, MEDLINE via OVID, EMBASE and The Cochrane Database (including the Central Register of Controlled Trials (CENTER)), updated to May 2024. The limitation to human studies was performed following the double negation strategy suggested by the Cochrane handbook, that is, combining the results with NOT (exp animals/not humans.sh.). A new search was performed in March 2025 to identify any potential new study.

Bibliographies of review articles on this topic and of all studied included for data extraction were screened and the database Web of Science was used to identify all the papers that cited the included papers.

In the attempt to include both published and unpublished data a specific theses database, www.theses.com/was searched and a hand search was performed for the last 2 years for the journals that published more about this topic and with a high impact factor (quartile 1—Q1) (Clinical Oral Implant Research, Journal of Clinical Periodontology, Journal of Dental Research, Journal of Periodontal Research, Clinical Oral Investigations and Clinical Implant Dentistry and Related Research). clinicaltrials.gov was searched to identify potential ongoing or already completed studies meeting the inclusion and exclusion criteria.

Any ambiguous or incomplete data were researched further by contacting the researchers responsible for the work.

No language restrictions were applied.

### 2.4 Study Selection and Data Extraction

A two‐stage screening was carried out. The first‐stage screening of titles and abstracts was carried out in duplicate and independently by two reviewers (P.E. and M.D.) to eliminate clearly irrelevant materials. This included reviews, in vitro studies, animal studies, case reports, and opinion articles. Disagreement was resolved by discussion. Any study where there was insufficient information in the title and abstract to make a clear decision as well as any study where there was a disagreement was included in the following stage of screening.

The second‐stage screening of the full text articles of all studies of possible relevance was also carried out independently and in duplicate by PE and MD. A data screening and abstraction form was devised at this stage to: (1) Verify the study eligibility derived from the above inclusion/exclusion criteria, (2) Carry out the methodological quality assessment, (3) Extract data on study characteristics and outcomes for the included studies.

Any disagreement was resolved by discussion and if necessary, a third reviewer (EC) was consulted.

Calculation and presentation of level of agreement at each of the two‐stage screening was carried out using Kappa statistics.

Data extraction was performed independently and in duplicate by two reviewers (P.E., M.D.). In case of missing or incomplete data and absence of further clarification by study authors, we excluded the report from the analysis.

### 2.5 Risk of Bias

Quality assessment of all included studies was conducted independently by two reviewers (SC, EC). The Risk of Bias In Non‐randomized Studies of Exposures (ROBINS‐E) tool was employed for prospective studies looking at the effect of smoking exposure over time (Higgins, Morgan, et al. 2024). Conversely, for retrospective studies, the Newcastle‐Ottawa scale was used, as this tool provides an easy quality assessment with the possibility to have a separate set of questions for case–control and cohort studies, with a score ranging from 1 (more biased) to 9 (https://www.ohri.ca/programs/clinical_epidemiology/oxford.asp).

### 2.6 Data Synthesis

Studies were grouped depending on the outcome and on the follow‐up period. The meta‐analyses were performed by using the software STATA 18.5 (StataCorp, College Station TX, USA) for the primary outcome (implant failure/survival both at patient‐ and implant‐level) and for crestal bone loss (CBL). Subgroup analysis was performed to assess differences among different time frames (1 year, 1–5 years, 5–10 years, > 10 years). Studies were assigned to the different time frames based on the mean follow‐up reported. For studies providing data on multiple follow‐ups, the longest follow‐up was considered, provided that at least 50% of the initial patients' data were presented. Data about implant failures and survival (dichotomous outcomes) in both exposed and not exposed groups were extracted and the meta‐analyses computed the Odds Ratio (OR) with its confidence interval by applying the DerSimonian and Laird's random effect method. Implant survival was used as a reference parameter in the meta‐analyses; hence an OR < 1 would indicate a significant negative impact of smoking. For CBL (continuous outcome), the value and its error measure (standard deviation, standard error, variance, or confidence interval) were extracted and the effect size was computed through the weighted mean method, by combining the results with the DerSimonian and Laird's random effect method and considering it significant for *p* < 0.05. The Cochran's test was adopted to evaluate heterogeneity, considering it significant for *p* < 0.01. Heterogeneity was investigated through *I* ^2^ statistics.

A sensitivity analysis was performed by including in the meta‐analysis also studies reporting the follow‐up period as mean ± SD, when SD was more than 10% of the mean value.

The appraisal of the quality of evidence was performed by using the Grading of Recommendations, Assessment, Development and Evaluation (GRADE) framework.

If multiple publications on the same population were identified, the study population was considered only once, extracting the data with the longest follow up.

### 2.7 Assessment of Reporting Biases

Publication bias was assessed by testing for funnel plot asymmetry, as described in the Cochrane Handbook (Higgins, Thomas, et al. 2024). If asymmetry was evident, it was investigated and the possible causes were described. Egger's test for small‐study effects was also performed in case > 10 studies were identified.

## 3 Results

### 3.1 Study Selection and Study Characteristics

Results are herein presented following the instructions of the Preferred Reporting Items for Systematic Review and Meta‐analysis (PRISMA) statement (Page et al. 2021).

**FIGURE 1:**
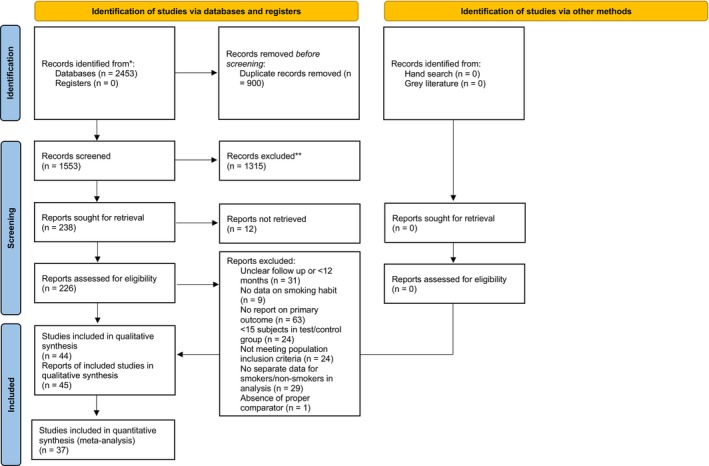
PRISMA flowchart describing the article selection process. Inter‐rater agreement at both steps of the selection process was > 0.9.

Forty‐five articles reporting on 44 studies (8 prospective, 35 retrospective studies and 1 study with an initial retrospective report (Vervaeke et al. 2012) followed by a long‐term prospective follow‐up report (Windael et al. 2020)) were included in the qualitative analysis (Figure 1), and their main characteristics are reported in Table 1. The largest study retrospectively investigated a cohort of 20,842 patients who received 50,333 dental implants (Chatzopoulos and Wolff 2023).

**TABLE 1:** Main characteristics of the included studies.

Study	Study design; country	Age; sex (M/F)	Number of patients; periodontal condition	Systemic health	Number of implants; location	Placement protocol; loading protocol; GBR	Restoration type	Smoking characteristics; comparator	Implant survival	Follow‐up
(Abduljabbar et al. 2018)	Retrospective (case–control); Saudi Arabia	S: 40.3 ± 2.5; NS: 42.6 ± 3.1; 56/0	56 (29S, 27NS); NR	Systemically healthy	91; S: 29 maxilla, 19 mandible, NS: 26 maxilla, 17 mandible in areas of missing premolars/M	Healed sites; CL; no GBR	Screw‐retained crowns	Cigarette smokers (smoked > 100 cigarettes in their life & presently smoked > 1 cigarette daily) for 8.9 ± 3.6 years; never used tobacco in any form	100% S and NS	S: 6.2 ± 0.1 years, NS: 6.1 ± 0.3 years
(Alhenaki et al. 2021)	Retrospective; Saudi Arabia	S: 58.5 ± 6.2; NS: 60.7 ± 5.6; 37/13	50 (25S, 25NS); attending SPC	Systemically healthy	82; maxillary tuberosity	Healed sites; CL; no GBR	72 screw‐retained, 10 cement retained crowns	Cigarette smokers for 8.7 ± 2.9 years; never smokers		5 years
(Agliardi et al. 2023)	Retrospective; Italy	57.3 ± 8.5; 80/93	173 (62S, 111NS); periodontitis history in 76 pts., all in regular SPC	Systemically healthy (19 controlled DM)	692; 288 maxilla and 404 mandible	Healed/post extraction; IL; no GBR	Full‐arch fixed prosthesis	Up to 20 cigarettes/day; non‐smokers		12–15 years
(Alahmari et al. 2019)^a^	Retrospective; Saudi Arabia	S: 44.5 ± 4.3; waterpipe users: 41.2 ± 4.7; NS: 43.3 ± 2.8; 123/0	123 (41S, 40 waterpipe users, 42NS); NR	Systemically healthy	123; S: 12 maxilla, 29 mandible, waterpipe users: 10 maxilla, 30 mandible, NS: 14 maxilla, 28 mandible	Healed bone; CL; NR	Cement‐retained crowns	S: ≥ 5 cigarettes/day for 5.5 ± 0.3 years; waterpipe users: inhale ≥ 1 daily for 10.6 ± 0.8 years; NS: non‐smokers who had not inhaled any form of tobacco for at least 1 year		S: 8.5 ± 0.3 years, waterpipe users: 8.6 ± 0.3 years, NS: 8.5 ± 0.5 years
(Al Amri et al. 2017)	Retrospective; Saudi Arabia	IL group S: 49.3 (35–53); NS: 40.7 (30–52) CL group S: 45.7 (33–53); NS: 41.3 (36–51); 61/0	61 (33S, 28NS); patients with several periodontal disease excluded. All patients in SPC	Systemically healthy	61 to replace mandibular premolars/M	Healed bone; 31 IL, 30 DL; no GBR	Screw‐retained crowns	Smoking ≥ 1 cigarette daily for ≥ 12 months. Mean numbers of cigarettes daily: IL 10.2 (10–20), DL 11.7 (8–20). Mean duration of smoking (years): IL: 14.7 (10–17), DL: 15.2 (11–20); never used tobacco in any form	100% S and NS	5 years
(Alazmi et al. 2021)^a^	Retrospective; Saudi Arabia	ES: 34.2 ± 1.3; NS: 35.1 ± 0.5; 92/35	127 (63S, 64NS); NR	Systemically healthy	157; ES: 40 maxilla, 35 mandible, NS: 42 maxilla, 40 mandible	NR; NR; NR	NR	Electronic cigarettes at least once daily for 9.3 ± 0.5 years. Mean duration: 6.5 ± 0.4 times per day, 3.5 ± 0.2 puffs per session; never used any form of nicotinic product	100% S and NS	ES: 8.8 ± 0.4 years, NS: 8.5 ± 0.2 years
(Alghamdi et al. 2020)	Prospective cohort study; Saudi Arabia	S: 45.5 ± 10.3; NS: 47.4 ± 9.4; 51/0	51 (26S, 26NS); NR	Systemically healthy	Unclear; posterior maxilla & mandible	Healed bone; IL; NR	NR	Having smoked ≥ 100 cigarettes in their life & currently smoking; duration of smoking 10.6 ± 0.4 pack years; never used tobacco in any form	100% S and NS	S: 6.2 ± 0.5 years, NS: 6.5 ± 0.3 years
(Alsaadi et al. 2008)	Retrospective; Belgium	NR; 172/240	412 (61S, 351NS); NR	Systemic diseases included	1514; 698 mandible (387 anterior), 816 jaw (413 anterior)	NR; NR; NR	NR	Three categories: < 10 cigarettes/day, 10–20 cigarettes/day or > 20 cigarettes/day; non‐smokers		2 years
(Alsahhaf et al. 2019)	Retrospective; Saudi Arabia	S: 40.4 ± 5.1 (TiZr), 44.5 ± 3.1 (Ti); NS: 45.6 ± 3.3 (TiZr), 43.7 ± 4.2 (Ti); 96/0	96 (48S, 48NS); NR	Systemically healthy	130 (66 TiZr, 64 Ti); maxillary/mandibular premolars	Healed bone; CL; no GBR	Cement‐retained crowns	Smoking at least 1 cigarette daily for ≥ 12 months; never used tobacco in any form	100% S and NS	3 years
(Balaguer et al. 2015)	Prospective cohort study; Spain	55.9 + −9.5 years; 40/55	95 (18S, 77NS); periodontal health of teeth in the remaining antagonist arch	Systemically healthy	360 implants supporting 107 overdentures; 136 maxillary, 224 mandibular	Healed bone; CL; no GBR	OVD	NR; non‐smokers	93%S, 97.1%NS	95 ± 20.3 months
(Bardis et al. 2023)	Retrospective; Romania	63.88 ± 11.71; 20/47	67 (48S, 130NS); 45 implants in patients with history of periodontal disease	Systemically healthy	178 (130NS, 48S); 91 maxilla, 87 mandible	Healed bone; CL; GBR in 88 implants	Fixed partial dentures	Smokers > 10 cigarettes/day; non‐smokers		7.89 ± 4.626 years
(Brizuela‐Velasco et al. 2021)	Retrospective; Spain	56.3 ± 11.8; unclear	110; periodontally healthy	90/200 had previous medical conditions	290; 157 maxilla, 133 mandible	34 immediate, 214 healed bone, 17 with sinus lift, 20 with simultaneous regeneration, 5 in previously regenerated bone; NR; GBR allowed	84 single crown, 155 fixed partial prosthesis, 51 OVD; 66 cemented and 174 screw‐retained	< 10 or > 10 cigarettes a day; non‐smokers		64.5 ± 11.7 months
(Cakarer et al. 2014)	Retrospective; Turkey	50.2 ± 13.21 years; 109/165	274		940 (246S, 694NS); 488 maxilla, 452 mandible	94 immediate, 846 healed bone; CL; bone augmentation in 57 implants	70 OVD, 712 fixed bridges, 158 single crowns	NR; non‐smokers		5 years
(Cavalcanti et al. 2011)	Retrospective; Italy	S: 47.1 (18–75) NS: 51.4 (17–85); 702/757	1727 (549S, 1178NS); 630 severe periodontitis. However, data available for 1477 (1019NS, 458S)	Systemically healthy	6720 (4460NS, 2260S) but data available for 6062 (3994NS, 2068S); unclear	Unclear; CL and IL; GBR as needed	OVD, fixed cross‐arch, partial fixed bridges, single crowns	NR; non‐smokers	94.5%S, 97.1%NS	5 years
(Castellanos‐Cosano et al. 2021)	Retrospective; Spain	< 40 years: 15, 41–55 years: 131, 56‐59 years: 25, > 70 years: 59 (implant level); 68/75	143 (16S, 115NS, 12 FS); 66 patients with history of periodontal disease	44 patients with systemic diseases and 52 taking medications	456 (67S, 346NS); 352 maxilla, 104mandible, 113 anterior, 343 posterior	NR; NR; 71 implants with regeneration	112 single crowns, 270 splinted crowns, 71 OVD, 3 unloaded; all screw‐retained	≥ 5 cigarettes per week in last 5 years; former smoker ≥ 5 cigarettes per week but stopped in the last 5 years; non‐smokers never used tobacco in any form	95.5%S, 96.5%NS, 90.7%FS	10 years
(Cha et al. 2014)	Retrospective; Korea	NR; 96/65	161 (18S, 143NS); periodontal health	Good systemic health/controlled medical conditions	462 (48S, 414NS); posterior maxilla	Healed bone; CL; sinus lift with xenogenic bone. In case of membrane perforation, porcine membrane and fibrin glue were also used	Screw‐retained fixed prostheses	≥ 1 cigarette/day; non‐smokers	85.42%S, 97.83%NS	57.1 ± 15.6 months
(Chatzopoulos and Wolff 2023)	Retrospective; USA	57.5 ± 14.27; 10,041/10798	20,482 (1673S, 18809NS); NR	Systemic diseases included	50,333; mandible and maxilla	NR; NR; NR	NR	NR; non‐smokers		83.86 ± 57.57 months
(Chrcanovic et al. 2017)	Retrospective; Sweden	≤ 30 years: 160, 31 ≤ 60 years: 385, > 60 years: 454; 479/520	999 (713NS, 263S, 23FS); NR	Systemic diseases included	3559 (2383NS, 1081S, 95FS); 489 anterior maxilla, 392 posterior maxilla, 255 anterior mandible, 326 posterior mandible	11 immediate placement; 20IL; 69 bone augmentations	NR	NR; non‐smokers and ex‐smokers	92.4%S, 96.4%NS, 88.4%FS	94.8 ± 78.7 months
(Deepa et al. 2018)	Retrospective; India	> 50 years: 85, < 50 years: 257; 150/202	352 (37S, 315NS); NR	110 in SSRI and 25 with DM (10 SSRI, 15 non‐SSRI)	680; NR	NR; NR; GBR whenever needed	NR	NR; non‐smokers		5 years
(Degidi et al. 2016)	Prospective cohort study; Italy	53.1 + −15.7 years; NR	114 (34S, 80NS) but at 10 years only 80 available; 32 periodontally treated	Systemically healthy	284 (88S, 196NS) but at 10 years only 193 available; NR	191 healed bone, 93 post‐extraction; IL; no GBR	Cement‐retained restorations	Current cigarette smokers and smokers that quit during study; non‐smokers, abstinence from smoking ≥ 5 years		10 years
(Doan et al. 2014)	Retrospective; Australia	54 (19–89); 216/256	472 (39S, 433NS); 87 pts. (186 implants) with persistent periodontitis, 32 pts. (64 implants) with destructive periodontitis	NR	1241; 652 maxilla, 589 mandible/357 incisors, 81 canines, 333 premolars, 470 M	743 healed (> 6 m), 373 (healed 3‐6 m), 125 immediate; 125 6–8 weeks, 256 10–12 weeks, 860 6 months; 83 GBR	958 single units, 283 fixed partial dentures	≥ 5 cigarettes/day; non‐smokers		10 years
(Garcia‐Bellosta et al. 2010)	Retrospective; Spain	55.4 ± 15.2; 138/185	323; patients in SPC/697 periodontitis (implant‐level) that received active therapy and were in SPC	Healthy and with systemic diseases	980 (380S, 600NS); maxilla and mandible	NR; CL; 59 sinus elevation	480 single crowns, 320 fixed partial denture, 180 in edentulous jaw	Light smokers: 1–10 cigarettes/day, moderate smokers: 11–20 cigarettes/day, heavy smokers: > 20 cigarettes/day; non‐smokers	96.4% ± 0.01S, 96.1% ± 0.01NS	48.6 (14.2–132.5) months
(Geurs et al. 2001)	Retrospective; USA	NR; NR	100; NR	NR	349 (62S); posterior maxilla (sinus graft)	Healed bone; NR; sinus graft (9 different graft materials)	NR	NR; non‐smokers	87.3%S, 95.2%NS	3.2 ± 1.25 years
(He et al. 2015)	Retrospective; China	44.9 (18–83 range); 704/673	1377; no periodontitis	No uncontrolled systemic diseases	2684 (388S, 2296NS); 412 anterior maxilla, 739 posterior maxilla, 122 anterior mandible, 1413 posterior mandible	Immediate: 71; healed bone: 1471; bone grafting surgeries included (GBR, sinus elevation)	Zirconia and gold alloy ceramic crowns	NR; non‐smokers		8 years
(Hong et al. 2020)	Retrospective; Korea	52.2 ± 10.5 native bone, 48.7 ± 10.7 regenerated bone; 134/106	240 (29S, 211NS)	No uncontrolled systemic diseases; 3 osteoporosis, 16 DM, 47 others	397 (48S); 43 anterior maxilla, 99 posterior maxilla, 14 anterior mandible, 241 posterior mandible	Healed bone and immediate; CL; 109 implants with simultaneous GBR, 24 implants in previously augmented bone	NR	NR; non‐smokers		33.8 ± 14 months for native bone, 30.6 ± 12 for regenerated bone
(Jesch et al. 2018)	Retrospective; Austria	57.7 ± 14.5 (women), 58 ± 14.6 (men); 3141/4642	7783 (9741S at 1 year); NR	Systemically healthy	18,945; 54.1% maxilla, 45.9% mandible	Healed bone and immediate; CL and IL (20.2%); sinus lifting, socket preservation, lateral block augmentation performed (193 implants)	Fixed (both cemented and screw‐retained) and removable prostheses	NR; non‐smokers	CSR implant‐level NS: 98.8% (1 year), 98.3% (3 years), 97.3% (5 years), 93.4% (10 years) Smokers: 97.4% (1 year), 95.1% (3 years), 93.9% (5 years), 88.3% (10 years) CSR patient‐level NS: 98.2% (1 year), 97.6% (3 years), 96.4% (5 years), 89.1% (10 years) S: 97.3% (1 year), 95% (3 years), 93.1% (5 years), 80.3% (10 years)	2.8 ± 3.2 up to 17.9 years
(Kandasamy et al. 2018)	Retrospective; India	47.5 (20–70); 88/112	200 (94S, 106NS); 10 patients (40 implants) with periodontal disease	No medically compromised but diabetes considered	650 (240S); 100 maxilla and 100 mandibles	NR; CL; 14 patients (62 implants) with augmentation	185 fixed (both screw‐retained and cemented) and 15 removable prostheses (OVDs)	NR; non‐smokers		8–15 years
(Koldsland et al. 2009)	Retrospective; Norway	43.8 (18–80); 40/69	109 (59S and FS; 50NS); 28 with history of periodontitis, SPC provided by their dentist	Systemic diseases included (17 cardiovascular disease, 5 DM)	372; maxilla and mandible	NR; loading between 1 to 25 months post insertion; NR	97 single crowns, 42 fixed partial prostheses, 20 fixed prostheses, 1 removable prostheses, 13 removable total prostheses	Smokers and former smokers combined; non‐smokers	84.7%S/FS, 98%NS	8.4 years (1.1 to 16)
(Lin et al. 2012)	Retrospective; USA	59.6; 32/43	75 (28S, 47NS); NR	Uncontrolled systemic diseases excluded	94NS, 62S; posterior maxilla (91 sinus grafting procedures)	NR; CL; sinus graft	NR	NR; non‐smokers	79%S, 87%NS	12 months
(Malo et al. 2015)	Retrospective; Portugal	58.9; 130/194	324 (79S, 245NS) (64 patients lost to follow up)	91 pts. with systemic conditions	1296; edentulous mandible anterior to the foramina	NR; IL; NR	Metal‐acrylic resin implant‐supported fixed prosthesis with a titanium framework	NR; non‐smokers		7 years clinical, 5 years radiographic
(Maló et al. 2018)	Retrospective; Portugal	53.7 ± 9.2 (53.2S ± 9.9S; 54.1NS ± 8.5NS); 81/119	200 (100S, 100NS) (9 dropouts); NR	49 with systemic disease (23S)	800; edentulous mandible, anterior to the mental foramina	Healed bone and immediate; IL; no GBR	Full‐arch fixed prostheses	Any cigarette smoking; no smoking habits	96.9%S, 99%NS	5 years
(Mangano et al. 2014)	Prospective cohort study; Italy	49.1 ± 11.5; 104/90	194 (35S, 159NS) (5 dropouts); active periodontal infections or other oral disorders excluded	Good systemic health	215; 124 posterior maxilla, 91 posterior mandible	Healed bone; CL; no GBR	Cemented single crowns	Patients who smoked cigarettes without considering the amount; non‐smokers	97.1%S, 98.7%NS	5.6 ± 2.7 years
(Mundt et al. 2006)	Retrospective; Germany	51.4; 65/94	159; NR	Systemic diseases included	663 (294NS, 115S, 247FS); 367 maxilla (191 anterior, 128 premolar, 48 M region), 296 mandible (98 M region)	NR; NR; NR	43 single crowns, 137 fixed partial dentures, 190 tooth/implant supported fixed partial dentures, 293 OVD	FS, and current S at the time of the follow‐up Examination; non‐smokers	85%S, 96%NS, 90%FS	88.2 months (43.6 to 146.3)
(Nitzan et al. 2005)	Retrospective; Israel	57 (23 to 89)	161 (102NS, 30 moderate S, 29 heavy S)	Systemically healthy; NR	646 (375 in S); mandible and maxilla	391 immediate; NR; no GBR or sinus lift	NR	Moderate S: ≤ 10 cigarettes/day, heavy S: > 10 cigarettes/day. Smokers also divided according to tobacco consumption: < 16 pack/years (PY) or > 16 PY; non‐smokers	87.8%S, 97.1%NS	42.9 months for S and 48.4 months for NS
(Peleg et al. 2006)	Prospective cohort study; USA	53 (42 to 81); 278/453	731 (226S, 505NS); good periodontal health	103 hypertension, 16 T1DM, 52 T2DM, 65 ischemic heart disease, 35 post MI	2132 (627S, 1505NS); posterior maxilla	Healed bone; CL; sinus elevation with graft	Fixed prostheses	NR; non‐smokers		4 to 7 years
(Raes et al. 2015)	Prospective cohort study; Belgium and USA	NS: 42 ± 18 for, S: 45 ± 15;42/43	85 (46S, 39NS); no untreated/uncontrolled periodontal disease	Uncontrolled systemic diseases excluded	85; anterior maxilla, between second premolars	Healed bone; IL; no GBR	Cemented crowns	10–30 cigarettes a day with a mean of 17; non‐smokers	98.96%S, 98.18%NS	2 years
(Rotim et al. 2021)	Retrospective; Croatia	46.5 (19–79); NR	670 (224S, 446NS); 170 with periodontal disease	27 T2DM, 3 T1DM, 5 T1DM + atherosclerosis, 45 > 1 disease, 625 none	1260; NR	NR; NR; NR	941 cemented, 319 screw‐retained	NR; non‐smokers		5 years
(Sánchez‐Pérez et al. 2007)	Retrospective; Spain	43.4 ± 15.4; NR but 11 men and 5 women had implant failures	No systemic or local contraindication to implant treatment	66 (23 light S, 11 moderate S, 6 heavy S, 26NS)	165 (94S, 71NS); 105 maxilla (65S), 65 mandible (29S)	NR; early loading (2 months post placement); no GBR	119 fixed, 46 OVD	Light S: < 10 cigarettes/day, moderate S: 10–20 cigarettes/day; heavy smokers: > 20 cigarettes/day; non‐smokers had never smoked or had quit ≥ 10 years ago and had not used any other form of tobacco	S < 10cig/day: 90.9%, S 10–20cig/day: 88%, S > 20cig/day: 69.2%, NS: 98.57%	5 years
(Sun et al. 2016)	Prospective cohort study; China	25–56; 32/0	32 (16S, 16NS); no periodontal disease before surgery	No acute/chronic systemic pathologies	45; posterior mandible	Healed bone; CL; NR	NR	Actively smoking ≥ 20 cigarettes a day for 10 years; never used any form of tobacco	100% S and NS	12 months
(Tawil et al. 2008)	Prospective cohort study; Lebanon	64.7 in T2DM, 59.6 in healthy; 57/33	90: (22S in T2DM, 18S in healthy); periodontal disease controlled before surgery, SPC every 6 months	45 T2DM	255 T2DM, 244 healthy; NR	NR; IL (58 in T2DM, 59 in healthy), CL (143 in T2DM, 142 in healthy); GBR: 20 in T2DM, 15 in healthy; lateral sinus lift: 33 in T2DM, 26 in healthy; internal sinus lift: 1 in T2DM, 2 in healthy	NR	NR; non‐smokers		42.2 months mean (1–12 years)
(Wach et al. 2023)	Retrospective; Poland	NR; NR	768; NR	Uncontrolled internal co‐morbidity excluded	2196; NR	NR; CL; no augmentation	NR	≥ 1 cigarettes; non‐smokers	93.26%S, 97.13%NS	5 years
(Windael et al. 2020, Vervaeke et al. 2012)	Prospective cohort study (although the 2‐year data presented as retrospective); Belgium	65.2 ± 11 (31–88); 48/73	121 (114 at year 1); patients with history of periodontitis not excluded	1DM, 1 taking bisphosphonates	453; (NS: 141 mandible, 228 maxilla, S: 35 mandible, 41 maxilla)	Healed bone; IL and CL; NR	67 single crowns, 180 fixed partial, 200 fixed cross‐arch bridges, 6 OVD	≥ 1 cigarette/day; ex‐smokers are included in non‐smokers	85.5%S, 94.2%NS	10 years
(Zhang et al. 2023)	Retrospective; China	57.5; 155/116 (prosthesis level)	257 (198NS, S 1–10 cigarettes: 53; S 11–20 cig: 28 at prosthesis level); patients without regular maintenance excluded	NR	1222; maxilla 114, mandible 157	NR; IL; GBR as necessary	271 full‐arch fixed prostheses (202 all‐on‐4, 69 all‐on‐6)	S 1–10 cigarettes/day, S 11–20 cigarettes/day. S of > 20 cigarettes excluded; non‐smoker	All‐on‐4: S 1–10cg/day: 93.9%, S 11–20 cig/day: 85.7%, NS 97.8% All‐on‐6: S 1–10cg/day: 92.6%%, S 11–20 cig/day: 93.7%, NS 96.7%	3–13 years
(Zuffetti et al. 2020)	Retrospective	51.6 ± 2.8; 99/75	174 (65S, 79NS, 30FS); 34 periodontitis; 6‐month recalls. Patients not following SPC excluded	Absence of medical conditions known as contraindications to implant surgery	254 (65S, 79NS, 30FS); posterior maxilla 94, mandible 80	Healed bone; CL; no GBR	135 screwed, 119 cemented prostheses	FS: those who had quit smoking for at least 5 years, S: current smokers; never smokers	95.4%S, 98.9%NS, 100%FS	41.9 months

Abbreviations: CL, conventional loading; DM, diabetes mellitus; ES, electronic cigarette smokers; FS, former smokers; IL, immediate loading; MI, myocardial infarction; NR, not reported; NS, non‐smokers; OVD, overdenture; S, smokers; SPC, supportive periodontal care; SSRI, selective serotonin reuptake inhibitors.

^a^ Studies that contributed to FQ2. Whenever implant survival was not directly reported in the studies, but only raw data on number of implants lost were indicated, these data were extracted and used (if possible) for meta‐analyses but they are not reported in the table.

14% of the studies (*n* = 6) recruited only male participants (Abduljabbar et al. 2018; Alahmari et al. 2019; Al Amri et al. 2017) (Alghamdi et al. 2020; Alsahhaf et al. 2019; Sun et al. 2016), while 86% (*n* = 38) included both female and male participants.

The great majority of the studies (*n* = 41) reported on the effect of cigarette smoking (FQ1), one study reported on both cigarettes and waterpipes (Alahmari et al. 2019), one on e‐cigarettes (Alazmi et al. 2021), and in one study they considered any type of cigarettes, without providing more specific details (Maló et al. 2018). Smoking habit and use of smokeless products was always self‐reported, with a significant heterogeneity between studies in terms of length of smoking and number of cigarettes smoked a day. Likewise, systemic health was heterogeneously reported, with some studies including only healthy participants and other studies recruiting patients with several comorbidities. The type of implant placement and loading protocols, as well as the type of implant‐supported restorations, varied between studies. While 59% of the studies did not consider or did not mention bone regenerative procedures in association to implant placement, 41% (*n* = 18) specifically reported that at least some cases received regenerative procedures, with 5 studies specifically focusing on sinus grafts (Cha et al. 2014; Geurs et al. 2001; Lin et al. 2012; Peleg et al. 2006).

### 3.2 Risk of Bias

**FIGURE 2:**
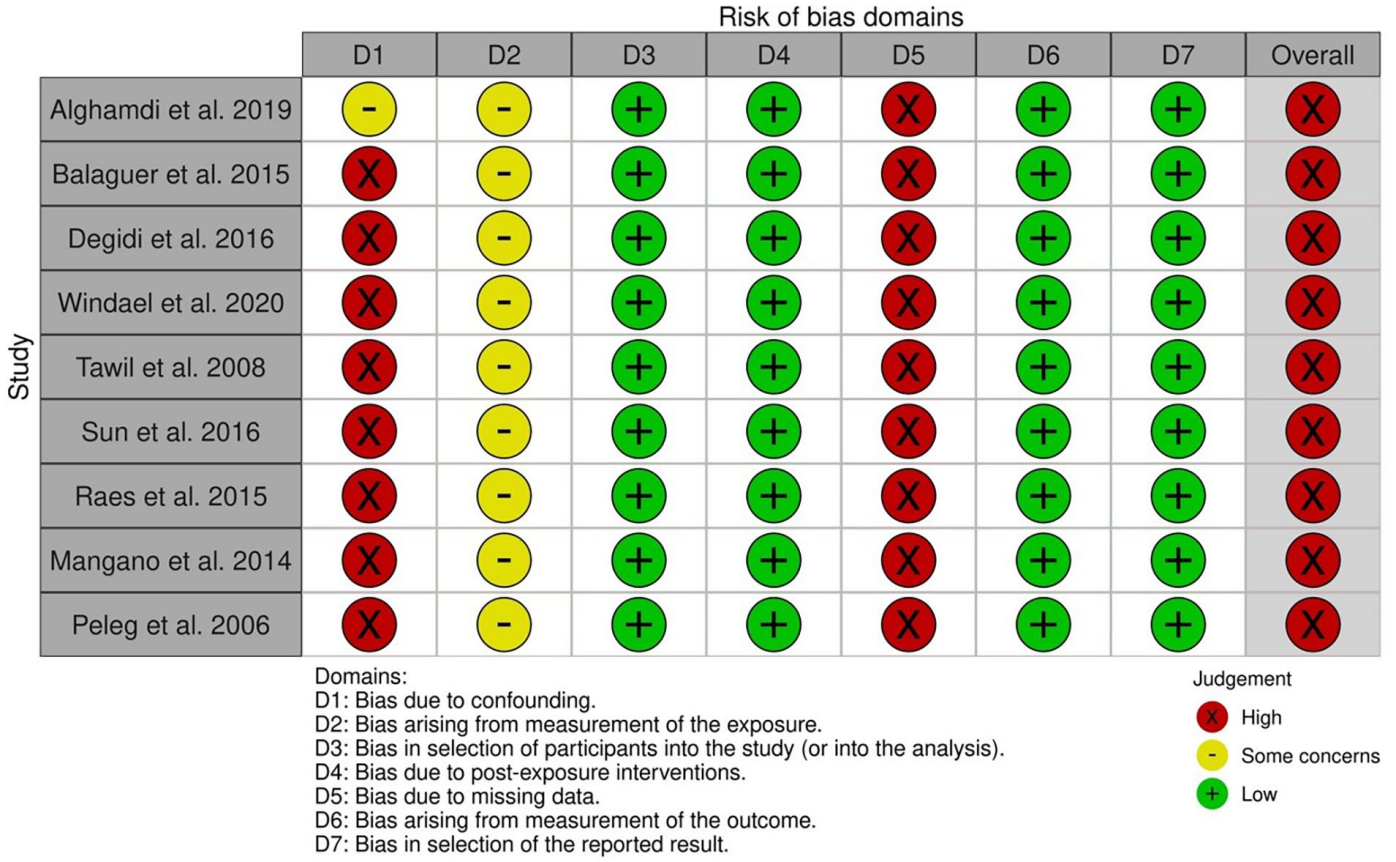
Risk of bias of prospective studies. Although the investigated population was the same in two articles, they were considered separately in the risk of bias assessment since one article retrospectively reported the 2‐year outcomes (Vervaeke et al. 2012) and one article prospectively reported the 10‐year outcomes (Windael et al. 2021).

The results of risk of bias evaluation are presented in Table 2 and Figure 2. For retrospective studies, the score ranged from 6 to 8, being downgraded due to the methods for assessing exposure (through self‐declaration) and the lack of control of confounding factors. For prospective studies, all the studies were considered at high risk of bias because of uncontrolled confounders and the lack of an objective assessment of smoke exposure.

**TABLE 2:** Risk of bias assessment of retrospective studies.

Cohort studies									
Study	Selection				Comparability	Exposure/outcome			Overall appraisal
*Cohort studies*									
	Representativeness of the exposed cohort Truly representative of the average _______________ (describe) in the community^a^ Somewhat representative of the average ______________ in the community^a^ Selected group of users, e.g., nurses, volunteers No description of the derivation of the cohort	Selection of the non exposed cohort Drawn from the same community as the exposed cohort^a^ Drawn from a different source No description of the derivation of the non exposed cohort	Ascertainment of exposure Secure record (e.g., surgical records)^a^ Structured interview^a^ Written self report No description	Demonstration that outcome of interest was not present at start of study Yes^a^ No	Comparability of cases and controls on the basis of the design or analysis Study controls for _______________ (Select the most important factor.)^a^ Study controls for any additional factor^a^ (This criteria could be modified to indicate specific control for a second important factor.)	Assessment of outcome Independent blind assessment^a^ Record linkage^a^ Self report No description	Was follow‐up long enough for outcomes to occur Yes (select an adequate follow up period for outcome of interest)^a^ No	Adequacy of follow up of cohorts Complete follow up—all subjects accounted for^a^ Subjects lost to follow up unlikely to introduce bias—small number lost — > ____ % (select An adequate %) (follow up, or description provided of those lost)^a^ Follow up rate < ____% (select an adequate %) and no description of those lost No statement	
*Case–control studies*									
	Is the case definition adequate? Yes, with independent validation^a^ Yes, e.g., record linkage or based on self reports No description	Representativeness of the cases a Consecutive or obviously representative series of cases^a^ a Potential for selection biases or not stated	Selection of Controls a Community controls^a^ b Hospital controls b No description	Definition of Controls a No history of disease (endpoint)^a^ c No description of source	Comparability of cases and controls on the basis of the design or analysis a Study controls for _______________ (Select the most important factor.)^a^ d Study controls for any additional factor^a^ (This criteria could be modified to indicate specific control for a second important factor.)	Ascertainment of exposure Secure record (e.g., surgical records)^a^ Structured interview where blind to case/control status^a^ Interview not blinded to case/control status Written self report or medical record only No description	Same method of ascertainment for cases and controls a Yes^a^ f No	Non‐response rate a Same rate for both groups^a^ b Non respondents described g Rate different and no designation	
(Abduljabbar et al. 2018)^a^	1	1	0	1	1	1	1	1	7
(Alhenaki et al. 2021)	1	1	0	1	1	1	1	1	7
(Agliardi et al. 2023)	1	1	0	1	0	1	1	1	6
(Alahmari et al. 2019)	1	1	0	1	1	1	1	1	7
(Al Amri et al. 2017)	1	1	0	1	1	1	1	1	7
(Alazmi et al. 2021)	1	1	0	1	1	1	1	1	7
(Alsaadi et al. 2008)	1	1	0	1	2	1	1	1	8
(Alsahhaf et al. 2019)	1	1	0	1	0	1	1	1	6
(Bardis et al. 2023)	1	1	0	1	1	1	1	1	7
(Brizuela‐Velasco et al. 2021)	1	1	0	1	1	1	1	1	7
(Cakarer et al. 2014)	1	1	0	1	1	1	1	1	7
(Cavalcanti et al. 2011)	1	1	0	1	1	1	1	1	7
(Castellanos‐Cosano et al. 2021)	1	1	0	1	1	1	1	1	7
(Cha et al. 2014)	1	1	0	1	0	1	1	1	6
(Chatzopoulos and Wolff 2023)	1	1	0	1	2	1	1	1	8
(Chrcanovic et al. 2017)	1	1	0	1	1	1	1	1	7
(Deepa et al. 2018)	1	1	0	1	0	1	1	1	6
(Doan et al. 2014)	1	1	0	1	0	1	1	1	6
(Garcia‐Bellosta et al. 2010)	1	1	0	1	2	1	1	1	8
(Geurs et al. 2001)	1	1	0	1	0	1	1	1	6
(He et al. 2015)	1	1	0	1	1	1	1	1	7
(Hong et al. 2020)	1	1	0	1	2	1	1	1	8
(Jesch et al. 2018)	1	1	0	1	1	1	1	1	7
(Kandasamy et al. 2018)	1	1	0	1	1	1	1	1	7
(Koldsland et al. 2009)	1	1	0	1	0	1	1	1	6
(Lin et al. 2012)	1	1	0	1	1	1	1	1	7
(Malo et al. 2015)	1	1	0	1	2	1	1	1	8
(Maló et al. 2018)	1	1	0	1	2	1	1	1	8
(Mundt et al. 2006)	1	1	0	1	1	1	1	1	7
(Nitzan et al. 2005)	1	1	0	1	1	1	1	1	7
(Rotim et al. 2021)	1	1	0	1	0	1	1	1	6
(Sánchez‐Pérez et al. 2007)	1	1	0	1	1	1	1	1	7
(Vervaeke et al. 2012)	1	1	0	1	1	1	1	1	7
(Wach et al. 2023)	1	1	0	1	1	1	1	1	7
(Zhang et al. 2023)	1	1	0	1	1	1	1	1	7
(Zuffetti et al. 2020)	1	1	0	1	1	1	1	1	7

^a^ Case–control design.

### 3.3 Primary Outcome

#### 3.3.1 FQ1

Only 9 studies directly calculated the OR/HR/RR of implant failure in tobacco smokers. Apart from three studies that did not find a significant interaction between implant failure and tobacco use (Garcia‐Bellosta et al. 2010; Alsaadi et al. 2008; Agliardi et al. 2023), the others overall reported an increased risk of implant failure in current smokers, also when adjusting for confounders like demographics, implant placement/loading protocols, surgical technique and area of the mouth (Appendix S2). A larger number of studies reported either the number of failed implants or the percentage of survived implants (Table 1), mainly at implant level. Implant survival in tobacco smokers ranged from 79% to 100%, while in non‐smokers it ranged from 87% to 100%.

An overall meta‐analysis not accounting for follow‐up time indicated a significantly reduced implant survival both at implant (36 studies) and patient (10 studies) level in cigarette smokers (OR = 0.40, 95% CI 0.27; 0.61, *p* < 0.001 and OR = 0.43, 95% CI 0.20; 0.90, *p* = 0.02, respectively), with high inter‐study heterogeneity at implant level (I^2^ = 78.81%) (Appendixes S3 and S4; and Figure 4). This means that cigarette smokers have a 2.5 times higher risk of implant failure at implant level and a 2.3 times higher risk of failure at patient level as compared with non‐smokers. Funnel plot (Appendix S5) and Egger's test did not show evidence for small‐study effects.

**FIGURE 3:**
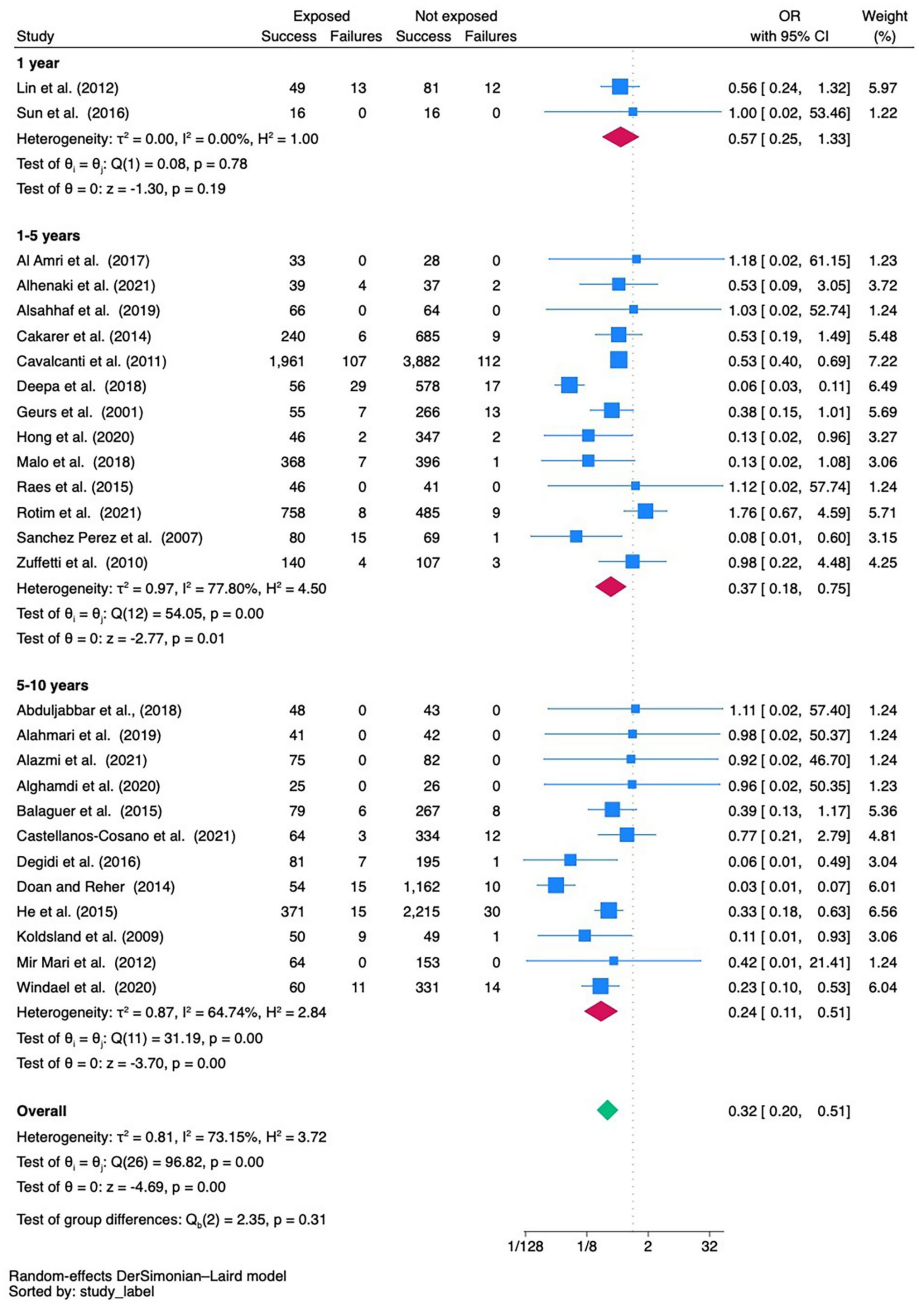
Meta‐analysis of implant failure data at implant level.

Meta‐analyses were then performed by grouping studies according to the follow‐ups. At 1 year, a non‐significant difference in implant survival at implant level (*p* = 0.19) in cigarette smokers compared with non‐smokers was indicated based on two studies (Figure 3).

**FIGURE 4:**
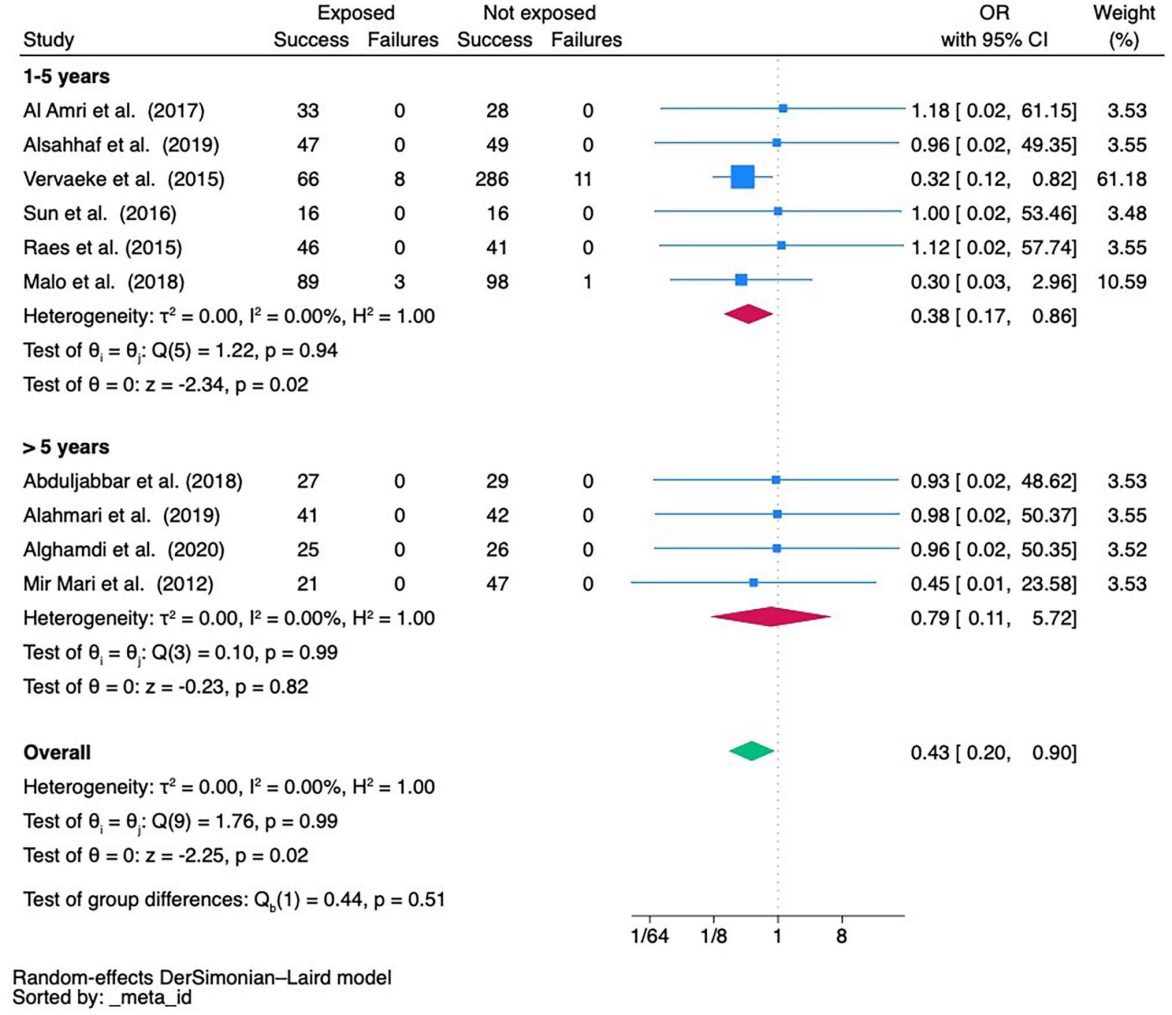
Meta‐analysis of implant failure data at patient‐level. An OR < 1 indicates a significant negative impact of smoking. Only 4 studies reported a follow‐up > than 5 years, but none reached 10 years of follow‐up.

A larger number of studies looked at the 1 to 5 years of follow‐up and the meta‐analyses at both implant (*n* = 13) and patient (*n* = 6) level indicated a reduced implant survival in cigarette smokers (OR = 0.37, 95% CI 0.18; 0.75, *p* = 0.01 and OR = 0.38, 95% CI 0.17; 0.86, *p* = 0.02, respectively), with a high inter‐study heterogeneity (*I* ^2^ = 77.80%) for the implant‐level data (Figures 3 and 4).

Likewise, meta‐analyses of studies reporting on 5 to 10 years of follow‐up confirmed a significant negative effect of cigarette smoking on implant survival at implant level (OR = 0.24, 95% CI 0.11; 0.51, *p* < 0.001) based on 12 studies, while the trend failed to reach significance at patient level, where only 4 studies with a follow‐up > 5 years could be included (OR = 0.79, 95% CI 0.11; 5.72, *p* = 0.82) (Figures 3 and 4).

Limited studies considered > 10 years of follow‐up, with Agliardi et al. (2023) failing to report a significant correlation between cigarette smoking and implant survival at 12–15 years and another large retrospective study (20,842 patients) over a 12‐year period also reporting a non‐significant effect of smoking on implant failure (Chatzopoulos and Wolff 2023). Conversely, in a study with up to 15 years of follow‐up Kandasamy et al. (2018) found that smoking significantly affected implant survival, with a 12.83% failure rate. Zhang et al. (2023) also reported that smoking had a negative effect on the 3 to 13‐year implant survival in patients receiving fixed full‐arch rehabilitations, with a significantly worse impact on all‐on‐four compared to all‐on‐six rehabilitations.

The quality of evidence was considered low both at patient‐ and implant‐level based on GRADE (Appendix S9).

Since only a minority of studies reported separate data for cigarette smokers and non‐smokers, it was not possible to assess the impact of demographics, systemic health, implant placement/loading protocols, and implant surface on implant survival. Likewise, it was not possible to assess whether smoking had a worse impact in cases where bone regeneration was performed.

#### 3.3.2 FQ2

A 100% implant survival was reported in the only study comparing e‐cigarette smokers (*n* = 63) to non‐smokers (*n* = 64) (Alazmi et al. 2021) and in the only study reporting on both cigarette and waterpipe smokers (*n* = 41 and 40, respectively) compared to non‐smokers (*n* = 42) (Alahmari et al. 2019), both with an 8‐year follow‐up.

### 3.4 Secondary Outcomes

Details about secondary outcomes can be found in S1.

#### 3.4.1 Radiographic Crestal Bone Loss (CBL)

##### 3.4.1.1 FQ1

A large number of studies (*n* = 18) radiographically compared CBL in cigarette smokers and non‐smokers (Appendix S6). When combining all studies together regardless of follow‐up, an increased CBL of 0.64 mm (95% CI 0.29; 0.99, *p* < 0.001) was found in smokers compared to non‐smokers (Appendix S7). At 1 to 5 years of follow‐up, meta‐analysis indicated an increased radiographic CBL of 0.10 mm (95% CI 0.03; 0.17, *p* < 0.001) in smokers, which increased in studies with > 5 years of follow‐up but without reaching statistical significance (mean 1.48, 95% CI −0.04; 3.01, *p* = 0.06). A high inter‐study heterogeneity was however present (*I* ^2^ = 57.29% and *I* ^2^ = 99.89%, respectively) (Appendix S7). The quality of evidence based on GRADE was considered as low (Appendix S9).

##### 3.4.1.2 FQ2

Alazmi et al. (2021) reported no differences in peri‐implant radiographic bone levels between e‐cigarette smokers and non‐smokers at up to 8 years of follow‐up. Conversely, one study indicated significantly worse CBL between cigarette (5.2 ± 0.3 mm mesially and 5.4 ± 0.2 mm distally) or waterpipe users (4.8 ± 0.2 mm mesially and 4.9 ± 0.3 mm distally) compared to non‐smokers (2.3 ± 0.1 mm mesially and 2.5 ± 0.2 mm distally) at 8 years of follow‐up, with almost double levels of peri‐implant bone resorption (Alahmari et al. 2019).

#### 3.4.2 Peri‐Implant Diseases and Other Biological Complications (Including Early Failure)

##### 3.4.2.1 FQ1

15 studies reported on peri‐implant disease incidence, although only 6 reported separate data for smokers and non‐smokers (Bardis et al. 2023; Hong et al. 2020; Maló et al. 2018; Peleg et al. 2006; Windael et al. 2020; Agliardi et al. 2023) (Appendix S8). Whilst one study explicitly reported no evidence of peri‐implant diseases at 3 and 5 years of follow‐up (Alsahhaf et al. 2019), the majority of the studies investigating the incidence of biological complications overall suggested an increased incidence of peri‐implantitis in cigarette smokers (Agliardi et al. 2023; Bardis et al. 2023; Maló et al. 2018; Windael et al. 2020), with Agliardi et al. (2023) reporting a tendency for higher risk of peri‐implantitis in mandibular implants not confirmed by other studies (Appendix S8). Remarkably, in a 10‐year retrospective study, smoking was associated with an increased risk of peri‐implantitis (OR = 6.11), being inferior only to history of periodontal disease (OR = 37.9) and implant site grafting (OR = 9.1) (Bardis et al. 2023).

Only limited studies provided separate data on early implant failure between non‐smokers and cigarette smokers (Appendix S8), without a clear trend of increased risk in the latter group (Mangano et al. 2014; Peleg et al. 2006; Maló et al. 2018; Kandasamy et al. 2018).

##### 3.4.2.2 FQ2

No data on biological complications could be found in the included studies in relation to smoke‐free users.

#### 3.4.3 Patient‐Reported Outcome Measures (PROMs)

None of the included studies investigated these outcomes.

#### 3.4.4 Changes in Peri‐Implant Crevicular Fluid (PICF) Markers and in Microbial Plaque Composition

None of the included studies investigated these outcomes.

## 4 Discussion

This systematic review suggests an increased risk of implant failure (both in the short and long term) and an increased radiographic CBL in cigarette smokers compared to non‐smokers. Insufficient data are available in relation to the effect of heated tobacco products, e‐vapors, and smoke‐free non‐inhalable products; hence, no conclusions can be drawn in this respect.

The outcomes on cigarette smokers are in line with the results of previous systematic reviews (Naseri et al. 2020; Chrcanovic et al. 2015) that combined study outcomes regardless of the different follow‐ups.

It is well established that tobacco smoking can impair the innate immune response via activation of the nuclear factor kappa B pathway and toll‐like receptors (Fatemi et al. 2013; Wu et al. 2014). Moreover, smoking negatively affects the osteogenic differentiation of mesenchymal stem cells and osteoblasts and promotes osteoclast formation by activating the RANKL signaling pathway, ultimately leading to bone destruction and reducing alveolar bone density (Xie et al. 2024). These mechanisms may account for the increased implant failure rate and CBL associated with smokers, as well as for the increased risk of biological complications such as peri‐implantitis. We were not able to assess the effect of smoking duration nor the possible dose effect on implant failure due to the paucity/heterogeneity of retrieved data reporting on these aspects. However, a previous review suggested that the success rate of dental implants is considerably reduced for patients smoking more than 10 cigarettes a day (Naseri et al. 2020). Owing to the paucity of studies reporting separate data for cigarette smokers and non‐smokers on different confounders, it was also not possible to clarify whether different placement and loading protocols should be considered at higher risk of implant failure in smokers and whether systemic health and concomitant medications may have an additive negative effect on smoking history.

Cigarette smoke has been previously reported to in vitro change the micromorphology and elemental composition of implant titanium surface due to the carbon‐containing compounds adsorption, which in turn influences the osteoblast‐titanium interactions, thus potentially jeopardizing implant osseointegration (Yang et al. 2019). Beside this mechanism, so far documented only in vitro, smoking induces vasoconstriction and reduces oxygenation of tissues, which are critical factors for osseointegration and bone formation (Ghanem et al. 2017). The limited data on early implant failure obtained from the studies included in the present systematic review (not designed to answer this question) do not allow us to confirm the hypothesis of a detrimental effect of smoking on osseointegration, although a recent systematic review reported an increased risk of early implant failure in smokers (OR 2.59, 95% CI 2.08; 3.23) (Fan et al. 2024).

Javed et al. (2019) hypothesized that nicotine and other chemicals in tobacco smoke could induce a state of oxidative stress in peri‐implant tissues, with raised levels of proinflammatory cytokines within the gingival crevicular fluid of smokers, leading to an increased likelihood of peri‐implant disease development through an inflammatory response. Nicotine was also shown to boost the virulence of oral microorganisms, possibly by stimulating the expression of virulence‐related genes or promoting increased biofilm formation (Wu et al. 2016; Rajasekaran et al. 2024). All the aforementioned mechanisms may explain the increased risk of peri‐implantitis in smokers confirmed also by the present systematic review, although a meta‐analysis could not be performed. It is interesting to note that, while the majority of long‐term studies indicated a negative effect of cigarette smoking on implant survival and CBL (Jesch et al. 2018; Windael et al. 2020; Kandasamy et al. 2018; Koldsland et al. 2009; Doan et al. 2014), few failed to confirm this finding (Abduljabbar et al. 2018; Tawil et al. 2008; Agliardi et al. 2023). This could be due to survivor bias, different supportive care protocols and level of oral hygiene of the patients, as well as to the presence of confounding factors like age and periodontitis history, that were not always accounted for.

While we followed stringent inclusion criteria, it is important to recognize that our review presents with some limitations. First of all, studies were extremely heterogenous in terms of population characteristics (e.g., age, gender, medical history, concomitant medications), implant placement/loading protocols, implant surface and on the definition of smoking status, with some studies involving only severe smokers and others including patients as long as they were smoking at least 1 cigarette a day (Table 1). Smoking habit was based on self‐reporting rather than on an objective measure in all studies, which resulted in a downgrade in the risk of bias assessment. As clearly reported in the literature, it is not uncommon for smokers to withhold their real smoking status or underreport its frequency/entity. According to a study, at least 1 in 10 smokers withholds their smoking status from healthcare providers (Curry et al. 2013), mainly because of the smoking‐related stigma and the social pressure around this unhealthy habit (Stuber and Galea 2009). Likewise, the comparator was not always clearly defined, with some studies including never‐smokers, other including currently non‐smoking patients and other that did not provide clear details (Table 1). Combining never‐smokers and former smokers is a limitation of the present review, as both the intensity of former smoking and the duration of the cessation period have shown to significantly influence the probability of tooth loss overtime (Ravida et al. 2020), and this can possibly translate also to implant loss.

The fact that in many of the selected studies smoking was not the main focus of the research and was investigated as a complicating factor involved in implant success (hence the retrospective/cohort study design) further increases the probability of introducing biases. Moreover, most of the studies reported implant‐based data rather than patient‐based data. Whenever possible we extracted both outcomes and performed separate meta‐analyses, but it should be recognized that implant‐based analyses carry the well‐known risk of not accounting for the patient effect. Due to all the aforementioned limitations, the quality of evidence based on GRADE was judged as low.

In the present review we calculated unadjusted OR for cigarette smoking and we could not perform any meta‐regression analysis due to the fact that only a few studies reported separate data for smokers and non‐smokers. Hence, our data did not consider other potentially important co‐factors influencing implant failure (such as the characteristics of the prosthesis, patient's compliance, hygienic parameters, periodontitis history, etc.). Interestingly, amongst the few studies that reported the adjusted OR/HR/RR (Appendix S2), the majority were in line with the outcomes of our meta‐analysis. This suggests that, whenever present, smoking exposure should be considered as one of the most important independent factors jeopardizing implant survival, accounting for more than a double risk of failures in exposed subjects. While the negative effect on CBL was significant (0.64 mm) and in line with previous reviews (Moraschini and Barboza 2016), its clinical relevance needs to be further elucidated. Moreover, our data suggest a higher risk of biological complications in smokers, with the majority of studies indicating a higher incidence of peri‐implantitis (Appendix S8). This is in agreement with a recent systematic review indicating a double risk of peri‐implantitis in smokers (Reis et al. 2023). We could not perform a meta‐analysis on the incidence of biological complications as the included studies were heterogeneous in terms of definition of peri‐implantitis, follow‐up time and unit of analysis (patient‐level vs. implant‐level data) and most studies did not report separate data between smokers and non‐smokers (Appendix S8). Remarkably, a 10‐year retrospective study suggested that the variable with the highest negative impact on peri‐implantitis development was history of periodontitis (OR = 37.9), followed by implant site grafting (OR = 9.1) and smoking (OR = 6.11) (Bardis et al. 2023).

Despite a number of studies are available in the literature on smoke‐free products and their impact on implant health, they could not be included in the present review due to the lack of reporting on the primary outcome. Remarkably, compared with traditional tobacco smoke, electronic cigarettes were reported to reduce or not change the clinical inflammatory symptoms of peri‐implantitis, such as bleeding on probing, probing depth, peri‐implant bone loss, and response to treatments. On the other hand, a wide range of oral health consequences (e.g., caries, oral cancer) may be associated with the use of e‐cigarettes (Fathi et al. 2024).

In conclusion, although dental implants can remain functionally stable in smokers and smoking should not be considered an absolute contraindication for implant therapy, it is imperative to recognize its detrimental effects on peri‐implant tissues. As such, individual behavioural counselling for smoking cessation should always be offered to patients receiving implant‐supported rehabilitation. Clinicians should clearly inform patients of the detrimental effects of smoking on the oral cavity and on the survival of implants (and teeth) and they should take into account patients' smoking status when planning supportive peri‐implant care recalls.

Future studies are suggested to investigate smoking as a continuous variable rather than a categorical one as this would allow to clarify the dose effect of smoking on implant survival and implant‐related complications. This would require also clarification of the smoking habit in terms of cigarettes per day or pack per years to enable meaningful dose–response analyses. It would also be advisable to validate self‐reported smoking status by the use of biochemical measures (e.g., exhaled carbon monoxide levels or urinary/blood/saliva cotinine assays or thiocyanate plasma/saliva/urinary levels), as already implemented in several medical studies (Hald et al. 2003; Benowitz et al. 2020). While we could not identify any study reporting on PROMs, microbiological changes and molecular changes in the peri‐implant crevicular fluid, in the future it would be interesting to gather such additional outcomes, which would likely allow to better profile smokers and stratify them based on their different risk profile.

Finally, future studies should report separate data for smokers and non‐smokers in terms of implant placement (and implant characteristics), loading protocols, bone regenerative procedures performed, demographics, history of periodontitis, concomitant systemic diseases, and patient's compliance, with the aim to clarify how these confounders/risk factors may differentially impact on the survival of dental implants and incidence of complications in smokers. As a matter of fact, all the aforementioned confounders may limit causal inference and it is therefore important that future studies control for them.

While the use of smokeless products such as heated tobacco, vapers and non‐inhalable smokeless tobacco has become extremely popular, evidence is still scarce on the effect of such habits on implant survival and risk of complications. Future studies are urgently needed to shade light on their impact on peri‐implant and periodontal tissues.

## Author Contributions

**Calciolari Elena:** conceptualization (equal), data curation (equal), methodology (lead), resources (equal), supervision (equal), visualization (equal), writing – original draft (equal). **Corbella Stefano:** conceptualization (equal), formal analysis (lead), methodology (equal), visualization (equal), writing – review and editing (equal). **Dourou Marina:** data curation (equal), methodology (equal), resources (equal), visualization (equal), writing – review and editing (equal). **Ercal Pinar:** data curation (equal), methodology (equal), visualization (equal), writing – review and editing (equal). **Donos Nikolaos:** conceptualization (equal), methodology (equal), supervision (supporting), writing – review and editing (equal).

## Funding

The authors have nothing to report.

## Ethics Statement

The authors have nothing to report.

## Consent

The authors have nothing to report.

## Conflicts of Interest

The authors declare no conflicts of interest.

## Supporting information


**Data S1:** Supporting Information.

## Data Availability

Data are available upon reasonable request.
