# Supplementary material for: Tobacco Smoking and Smoke‐Free Products as Risk Factors for Dental Implants: A Systematic Review
Source: Clin Oral Implants Res. 2026 Mar 3;37(3):262–86. doi: 10.1111/clr.70108 (PMC12975700; doi:10.1111/clr.70108)
Supplement: Supplementary file 1 — Data S1: Supporting Information. [file CLR-37-262-s001.docx]

**Appendix 1** Details about the search strategy

**MEDLINE via OVID (1946 to 02^nd^ May 2024)**

|  | MeSH term | Free-Text search |
| --- | --- | --- |
| Population | Dental implants/ OR exp Dental implantation | “Dental implant*” OR “Surgical Dental Prosthes$s” |
| Exposure/comparison | Exp smoking OR exp smoking devices OR nicotine | Smoking OR tobacco OR “non-tobacco product” OR cigar or cigarette OR vaping or e-cigarette or pipe OR hookah OR (tobacco and (chewing OR dip OR dissolvable OR snuff OR snus)) OR “heated tobacco” OR shisha OR sheesha OR waterpipe OR narghile |

Filter for HUMAN STUDIES:

NOT (Exp animals/not humans.sh)

Number of hits: 1093

**EMBASE**

|  | Emtree term | Free-Text search |
| --- | --- | --- |
| Population | tooth implant/exp | “Dental implant*” |
| Exposure/comparison | smoking/exp OR smoking device/exp OR tobacco/exp OR nicotine/exp | Smoking OR tobacco OR “non-tobacco product” OR cigar or cigarette OR vaping or e-cigarette or pipe OR hookah OR (tobacco and (chewing OR dip OR dissolvable OR snuff OR snus)) OR “heated tobacco” OR shisha OR sheesha OR waterpipe OR narghile |

Filter to exclude animal studies:

NOT ([animals]/lim NOT [humans]/lim)

Number of hits: 1198

CENTRAL

|  | MeSH term | Free-Text search |
| --- | --- | --- |
| Population | Dental implants/ OR exp Dental implantation | Dental implant OR Surgical Dental Prosthes* |
| Exposure/comparison | Exp smoke OR exp smoking devices OR nicotine OR tobacco use | Smoking OR tobacco OR non-tobacco product OR cigar or cigarette OR vaping or e-cigarette or pipe OR hookah OR (tobacco and (chewing OR dip OR dissolvable OR snuff OR snus)) OR “heated tobacco” OR shisha OR sheesha OR waterpipe OR narghile |

Number of hits: 162 trials

**Appendix 2 Details on implant failure in smokers expressed as RR/OR/HR in the included studies.** **GEE: General estimating equations, ARP: Alveolar ridge preservation, OR: Odds Ratio, HR: Hazard Ratio, RR: Risk Ratio, aHR: adjusted Hazard Ratio.**

| **Author & Year** | **Analysis** | **Confounders / Adjustments** | **Results** |
| --- | --- | --- | --- |
| (Agliardi et al., 2023) | Cox regression analysis |  | HR= 0.551 (0.120-2.536) p=0.444 |
| (Alsaadi et al., 2008) | GEE Univariate logistic regression | Implant related, systemic and behavioural factors, local bone factors | <10 cigarettes OR: 1.39 (0.38 – 5.09)  10-20 cig. OR: 2.92 (0.97 – 8.77)  >20 cig. OR 1.21 (0.39 – 3.73).  P=0.28/adjusted p=1 |
| (Chrcanovic et al., 2017) | Cox regression analysis | - | Current HR: 2.363 (1.326 – 4.180) p=0.003  Former HR: 2.302 (0.514 – 10.306) p=0.276 |
| (Garcia-Bellosta et al., 2010) | Multivariate Cox regression analysis | Periodontitis, n° of implants, implant diameter, implant length, sinus elevation | HR: 0.91 (*p* = 0.806) |
| (He et al., 2015) | Multivariate Cox regression analysis |  | OR: 2.277 (1.087-4.774) p=0.029 |
| (Jesch et al., 2018) | Multivariate Cox regression analysis | Sex, Age > 65 yrs, Jaw, Region, Loading protocol, Surgical technique | Patient-level HR: 2.1 (1.5 – 2.9) p<0.001  Implant-level HR: 2.3 (1.6 – 3.4) p<0.001 |
| (Malo et al., 2015) | Univariete Cox regression analysis |  | HR: 2.32 (0.81 – 6.69), p=0.119 |
|  | Multivariate Cox regression | Age, gender, opposing dentition, bisphosphonate therapy | HR: 5.28 (1.33-20.91) p=0.02 |
| (Mundt et al., 2006) | Multivariate Cox regression analysis | Gender, Age, Region, Prosthesis | HR: 1.04 (1.01 – 1.07) p=0.004 |
| (Zhang et al., 2023) | Cox proportion  hazards models |  | HR:1.7 (1-2.8) p=0.048 in all-on-4 vs all-on-6 |

**Appendix 3** Meta-analysis of implant failure considering all studies regardless of follow-up and reporting data at implant level and associated Galbraith plot.


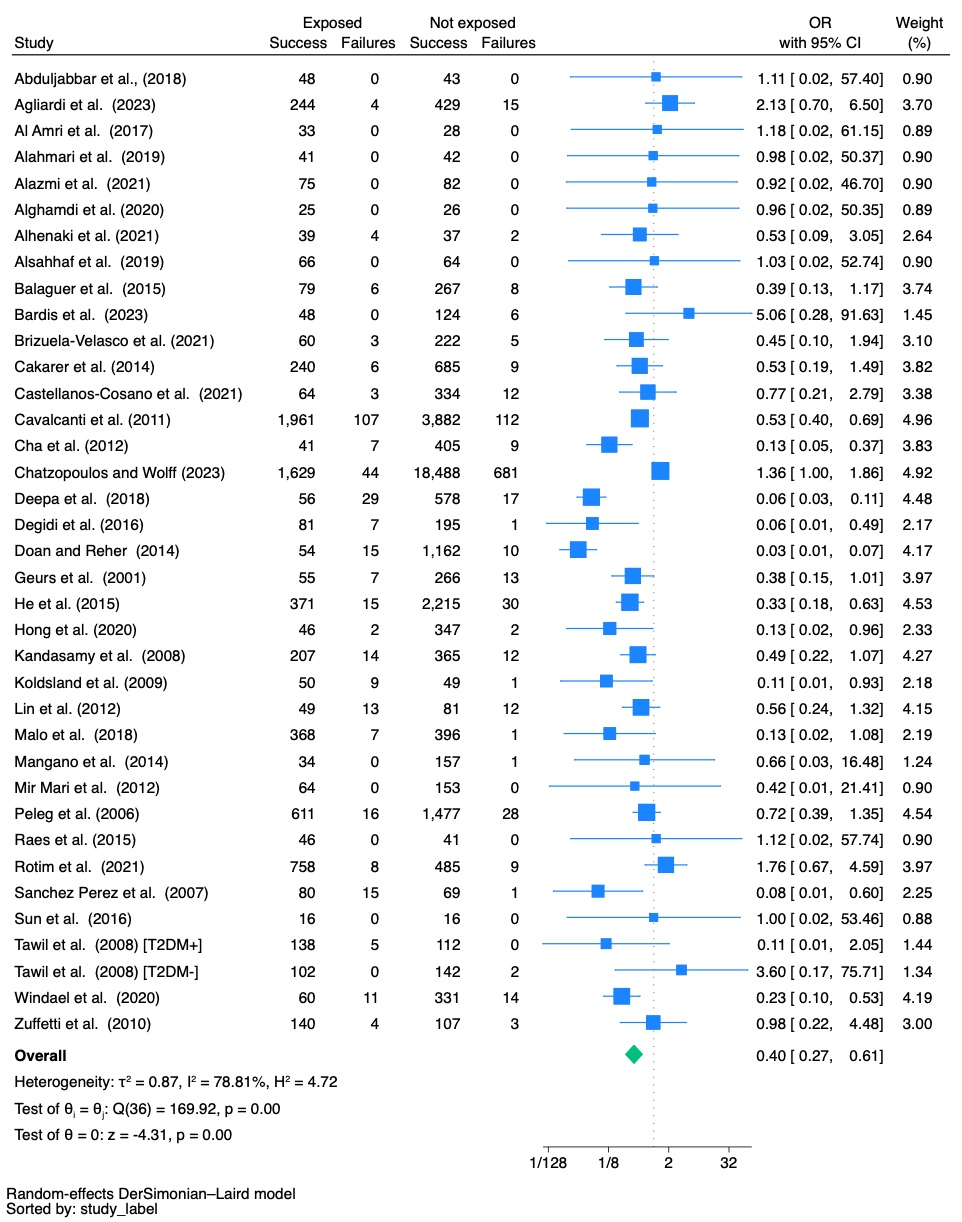


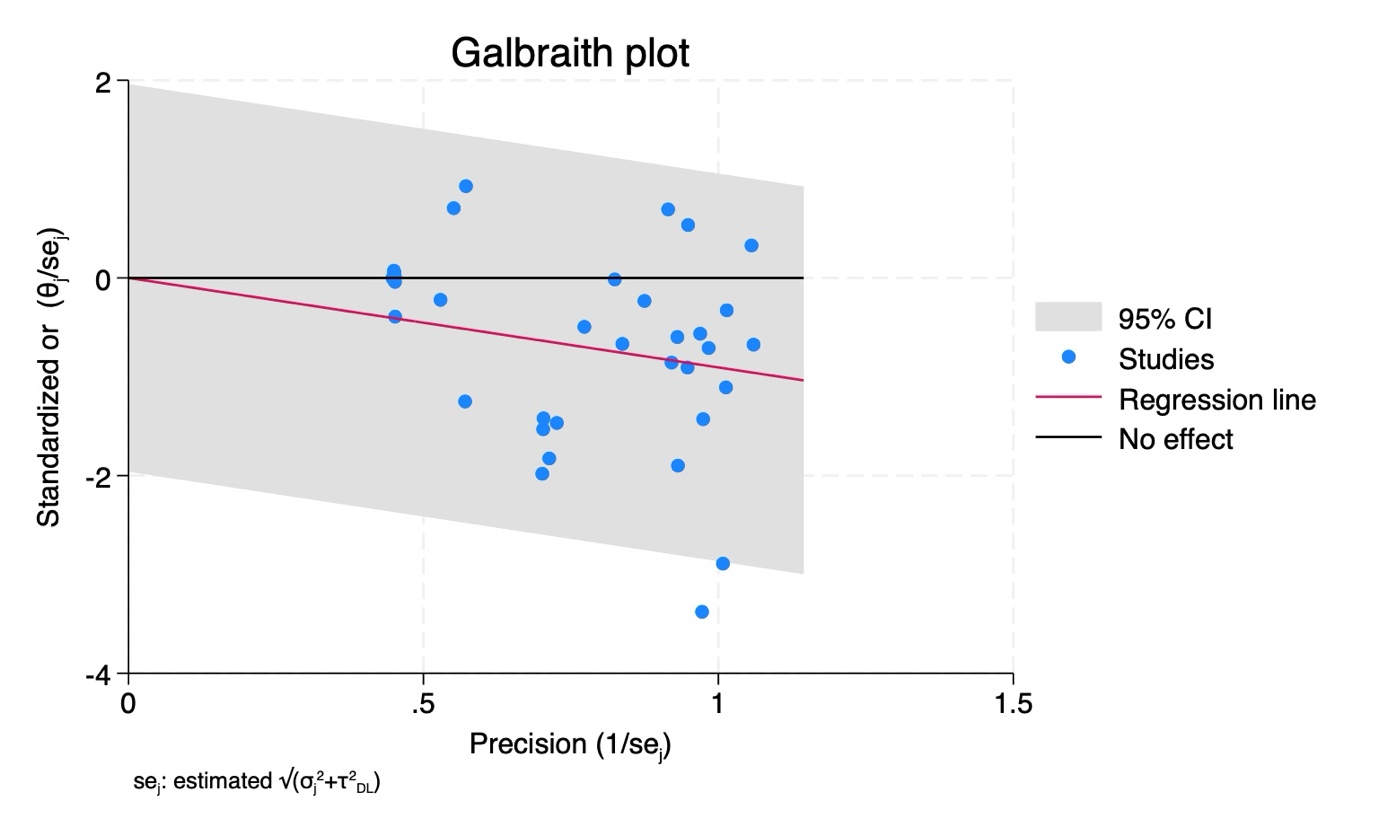


**Appendix 4** Galbraith plot related to the meta-analysis of implant failure considering all studies reporting data at patient- level, regardless of follow-up


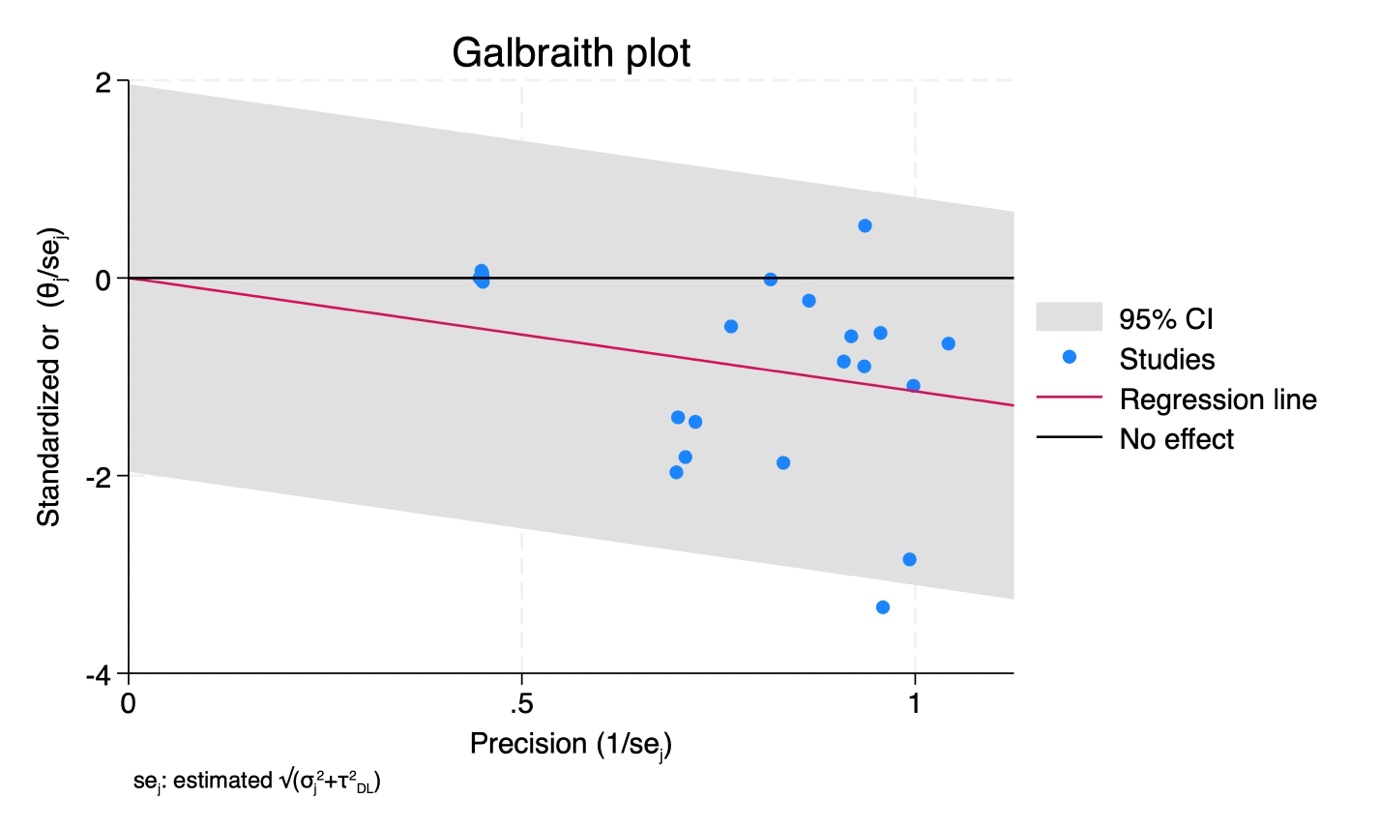


**Appendix 5** Funnel plot for mean the risk of implant failure in smokers compared with non-smokers.

**
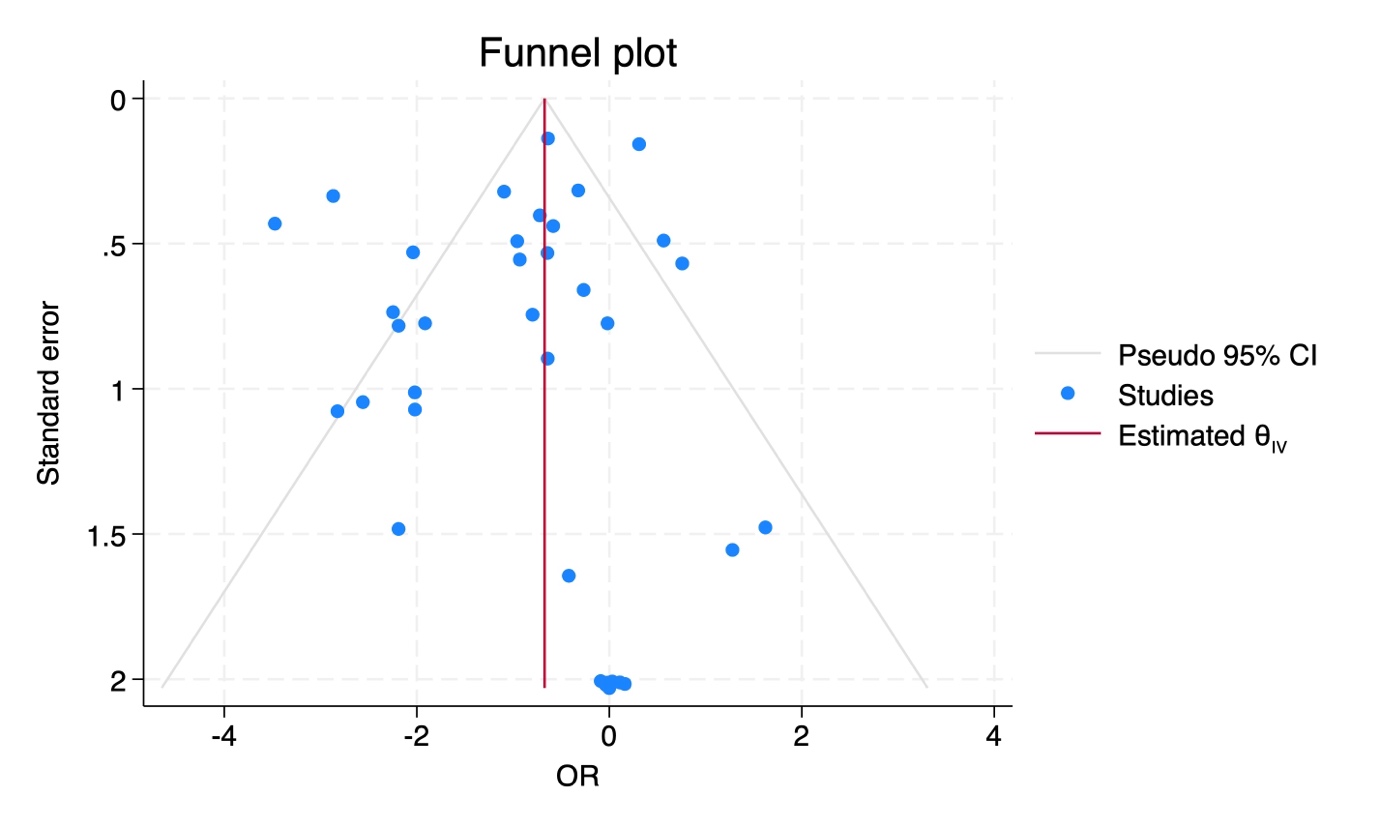
**

**Appendix 6** Summary of crestal bone loss (CBL) outcomes reported by the included studies.

| **Study** | **Crestal Bone Loss (mm)**  **Smokers** | **Crestal Bone Loss (mm)**  **Non-smokers** |
| --- | --- | --- |
| (Abduljabbar et al., 2018) | S: 6.2±0.1yrs  Mesial: 1.4±0.5 Distal: 1±0.2  *Adjusted for jaw location:*  Maxilla:  Mesial: 1.7±0.1 Distal: 1.5±0.2  Mandible:  Mesial: 1.2±0.2 Distal: 0.8±0.1 | NS: 6.1±0.3yrs  Mesial: 1.1±0.2 Distal: 1.2±0.1  *Adjusted for jaw location:*  Maxilla:  Mesial: 1.3±0.4 Distal: 1.4±0.2  Mandible:  Mesial: 0.8±0.1 Distal: 1±0.3 |
| (Alhenaki et al., 2021) | 12 months  S:  Mean: 0.27 (0.20-0.38) Mesial: 0.16 (0-0.26) Distal: 0.49 (0.31-0.85)  60 months  S:  Mean: 0.41 (0.2-0.49) Mesial: 0.30 (0-0.39) Distal: 0.68 (0.4-0.99) | 12 months  NS:  Mean: 0.16 (0-0.3) Mesial: 0.15 (0-0.25) Distal: 0.28 (0.1-0.64)  60 months  NS:  Mean: 0.25 (0.1-0.45) Mesial: 0.23 (0-0.42) Distal: 0.34 (0.21-0.66) |
| (Alahmari et al., 2019)* | S: 8.5±0.3yrs  Mesial: 5.2±0.3 Distal: 5.4±0.2  WS: 8.6±0.3yrs  Mesial: 4.8±0.2 Distal: 4.9±0.3 | NS: 8.5±0.5yrs  Mesial: 2.3±0.1 Distal: 2.5±0.2 |
| (Al Amri et al., 2017) | 5yrs  Immediate Loaded  S:  Total: 3.5(0.6-4.6) Mesial: 3.2(0.8-4.1) Distal: 3.7(0.6-4.6)  Delayed Loaded  S:  Total: 4.1(0.4-4.4) Mesial: 4.4(0.4-3.9) Distal: 3.8(0.6-4.4) | 5yrs  Immediate Loaded  NS:  Total: 0.6(0-1.2) Mesial: 0.5(0-0.8) Distal: 0.8(0.3-1.2)  Delayed Loaded  NS:  Total: 0.5(0-1.5) Mesial: 0.4(0-0.8) Distal: 0.6(0.4-1.5) |
| (Alazmi et al., 2021)* | S: 8.8±0.4yrs  Mesial: 1.4±0.0.4 Distal: 1.3±0.05  *Adjusted for jaw location:*  Maxilla:  Mesial: 1.7±0.07 Distal: 1.5±0.05  Mandible:  Mesial: 1.3±0.1 Distal: 1.1±0.06 | NS: 8.5±0.2yrs  Mesial: 1.05±0.06 Distal: 1.2±0.07  *Adjusted for jaw location:*  Maxilla:  Mesial: 1.4±0.08 Distal: 1.5±0.07  Mandible:  Mesial: 0.9±0.005 Distal: 1±0.004 |
| (Alghamdi et al., 2020) | S: 6.2±0.5yrs  Mesial: 4.4±1.05 Distal: 4.3±0.9 | NS: 6.5±0.3yrs  Mesial: 1.58±0.2 Distal: 1.55±0.1 |
| (Alsahhaf et al., 2019) | 3yrs  Titanium  S:  Mean: 1.35 Mesial: 1.30 Distal: 1.40  Titanium-Zirconium  S:  Mean: 1.26. Mesial: 1.20 Distal: 1.30  5yrs  Titanium  S:  Mean: 1.50 Mesial: 1.50 Distal: 1.50  Titanium-Zirconium  S:  Mean: 1.53 Mesial: 1.50 Distal: 1.60 | 3yrs  Titanium  NS:  Mean: 1.08 Mesial: 1.10 Distal: 1.00  Titanium-Zirconium  NS:  Mean: 1.18 Mesial: 1.10 Distal: 1.20  5yrs  Titanium  NS:  Mean: 1.30 Mesial: 1.30 Distal: 1.30  Titanium-Zirconium  NS:  Mean: 1.40 Mesial: 1.40 Distal: 1.40 |
| (Castellanos-Cosano et al., 2021) | 10yrs ; MBL per year (all implants)  S: 0.39±0.52 | 10yrs ; MBL per year (all implants)  FS: 0.29±0.30  NS:0.20±0.29 |
| (Degidi et al., 2016) | 6 months  S: 0.50±0.16  12 months  S: 0.91±0.17  24 months  S: 1.09±0.19  36 months  S: 1.25±0.23  48 months  S: 1.39±0.27  60 months  S: 1.51±0.28  72 months  S: 1.64±0.31  84 months  S: 1.74±0.32  96 months  S: 1.84±0.33  108 months  S: 1.88±0.30  120 months  S: 1.94±0.29 | 6 months  NS: 0.42±0.14  12 months  NS: 0.81±0.20  24 months  NS: 1.00±0.21  36 months  NS: 1.16±0.23  48 months  NS: 1.27±0.27  60 months  NS: 1.38±0.28  72 months  NS: 1.49±0.31  84 months  NS: 1.57±0.31  96 months  NS: 1.66±0.32  108 months  NS: 1.76±0.34  120 months  NS: 1.81±0.35 |
| (Maló et al., 2018) | 5yrs  S: 1.98±1.02  *Adjusted for implant location:*  Anterior  S: 1.85±1.28  Posterior  S: 2.11±1.37 | 5yrs  NS: 1.68±0.76  *Adjusted for implant location:*  Anterior  NS: 1.66±0.94  Posterior  NS: 1.71±0.78 |
| (Nitzan et al., 2005) | 3.8yrs (1-7yrs range)  Average:  S: 0.153±0.092  *Adjusted for jaw location:*  Maxilla:  MS: 0.1233±0.15559 HS: 0.1897±0.18247  Mandible:  MS: 0.1502±0.15867 HS: 0.1390±0.15990 | 3.8yrs (1-7yrs range)  Average:  NS: 0.047±0.048  *Adjusted for jaw location:*  Maxilla:  NS:0.0460±0.07035  Mandible:  NS: 0.0487±0.09115 |
| (Raes et al., 2015) | 2yrs  S: 0.22±0.42 (median 0.20) | 2yrs  NS: -0.33±1.19 (median -0.35) |
| (Sánchez-Pérez et al., 2007) | 5yrs (MBL1 at functional loading)  S: 2.71±1.55  5yrs (MBL2 at most recent follow up or failure)  S: 2.41±1.46  5yrs (bone gain MBL2-MBL1)  S: -0.07±0.97 | 5yrs (MBL1 at functional loading)  NS: 2.78±1.50  5yrs (MBL2 at most recent follow up or failure)  NS: 3.13±1.59  5yrs (bone gain MBL2-MBL1)  NS: 0.24±1.4 |
| (Sun et al., 2016) | 12 months  HS: 2.08±1.08 | 12 months  NS: 0.9±1.10 |
| (Wach et al., 2023) | Baseline  S: 0±1.13  5yrs  S: 0.42±1.32 | Baseline  NS:0±0.85  5yrs  NS: 0±1.25 |
| (Windael et al., 2020) (Vervaeke et al., 2012) | 31±7.15 months (24-58 months range)  Implant level  *Adjusted for jaw location:*  Maxilla + Mandible:  S: 0.53±0.92 (0-5.9)  Mandible:  S: 0.25±0.5 (0-4.9)  Maxilla:  S: 0.74±1.07 (0-5.9)  10yrs (post-op bone loss)  *Adjusted for amount of bone loss:*  bone loss > 1 mm:  S: 40.3% ; mandible (n=5) (17.9%), maxilla (n=20) (58.8%)  bone loss ≤ 1 mm:  S: 59.7% ; mandible (n=23) (81.2%), maxilla (n=14) (41.2%)  bone loss > 2 mm:  S: 30.6% ; mandible (n=3) (10.7%), maxilla (n=16) (47.1%)  bone loss ≤ 2 mm:  S: 69.4% ; mandible (n=25) (89.3%), maxilla (n=18) (52.9%) | 31±7.15 months (24-58 months range)  Implant level  *Adjusted for jaw location:*  Maxilla + Mandible:  NS: 0.29±0.54 (0-7.1)  Mandible:  NS: 0.22±0.5 (0-4.55)  Maxilla:  NS: 0.33±0.65 (0-7.1)  10yrs (post-op bone loss)  *Adjusted for amount of bone loss:*  bone loss >1mm:  NS: 18.8% ; mandible (n=19) (15.1%), maxilla (n=44) (21.1%)  bone loss ≤ 1 mm:  NS: 81.2% ; mandible (n=107) (84.9%), maxilla (n=165) (78.9%)  bone loss > 2 mm:  NS: 11.3% ; mandible (n=14) (11.1%), maxilla (n=24) (11.5%)  bone loss ≤ 2 mm:  NS: 88.7% ; mandible (n=112) (88.9%), maxilla (n=185) (88.5%) |
| (Zhang et al., 2023) | All-on-4  1yr  S: 0.5 (0.8) - 1-10 cigarettes/day; 0.7 (1.0) - 11-20 cigarettes/day  5yrs  S: 0.7 (1.1) - 1-10 cigarettes/day; 0.9 (1.1) - 11-20 cigarettes/day  10yrs  S: 1.1 (0.9) - 1-10 cigarettes/day; 1.3 (1.2)- 11-20 cigarettes/day  All-on-6  1yr  S: 0.4 (1.0) - 1-10 cigarettes/day; 0.5 (0.9)- 11-20 cigarettes/day  5yrs  S: 0.8 (1.3) - 1-10 cigarettes/day; 0.8 (0.7) - 11-20 cigarettes/day  10yrs  S: 0.8 (0.5) - 1-10 cigarettes/day; 0.9 (1.1) - 11-20 cigarettes/day | All-on-4  1yr  NS: 0.4 (0.8)  5yrs  NS: 0.6 (0.9)  10yrs  NS: 0.6 (0.8)  All-on-6  1yr  NS: 0.5 (0.6)  cigarettes/day  5yrs  NS: 0.6 (0.8)  10yrs  NS: 0.6 (0.9) |
| (Zuffetti et al., 2020) | 41.9 months  S:  Mesial: 0.24±0.4 Distal: 0.23±0.3 | 41.9 months  NS:  Mesial: 0.19±0.6 Distal: 0.18±0.7 |

S – smokers; NS – non-smokers; CS – cigarette smokers; WS – waterpipe smokers; FS – former smokers; MS – moderate smokers; HS – heavy smokers

**Appendix 7** Meta-analysis on marginal crestal bone loss (CBL) and associated Galbraith plot.


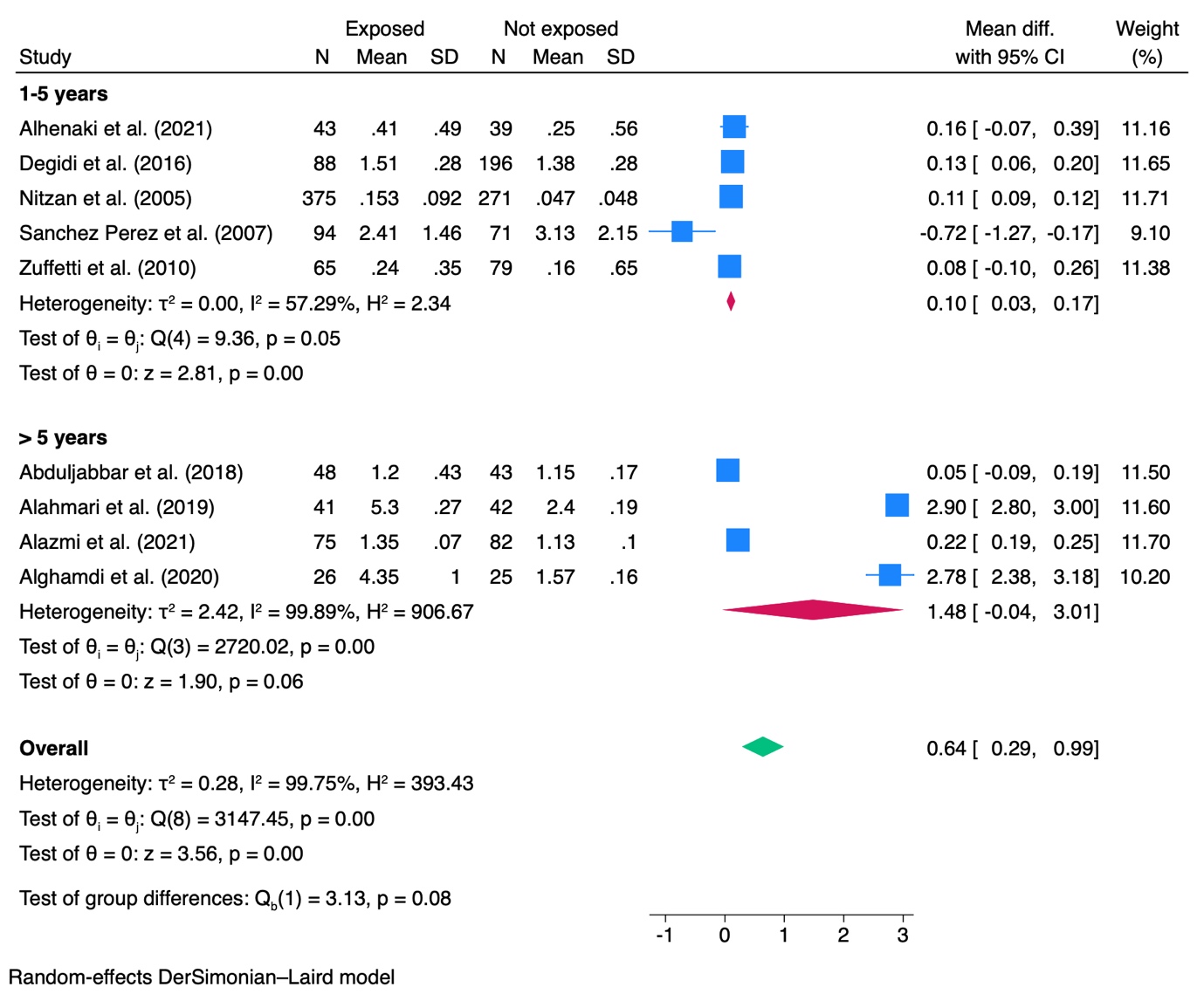


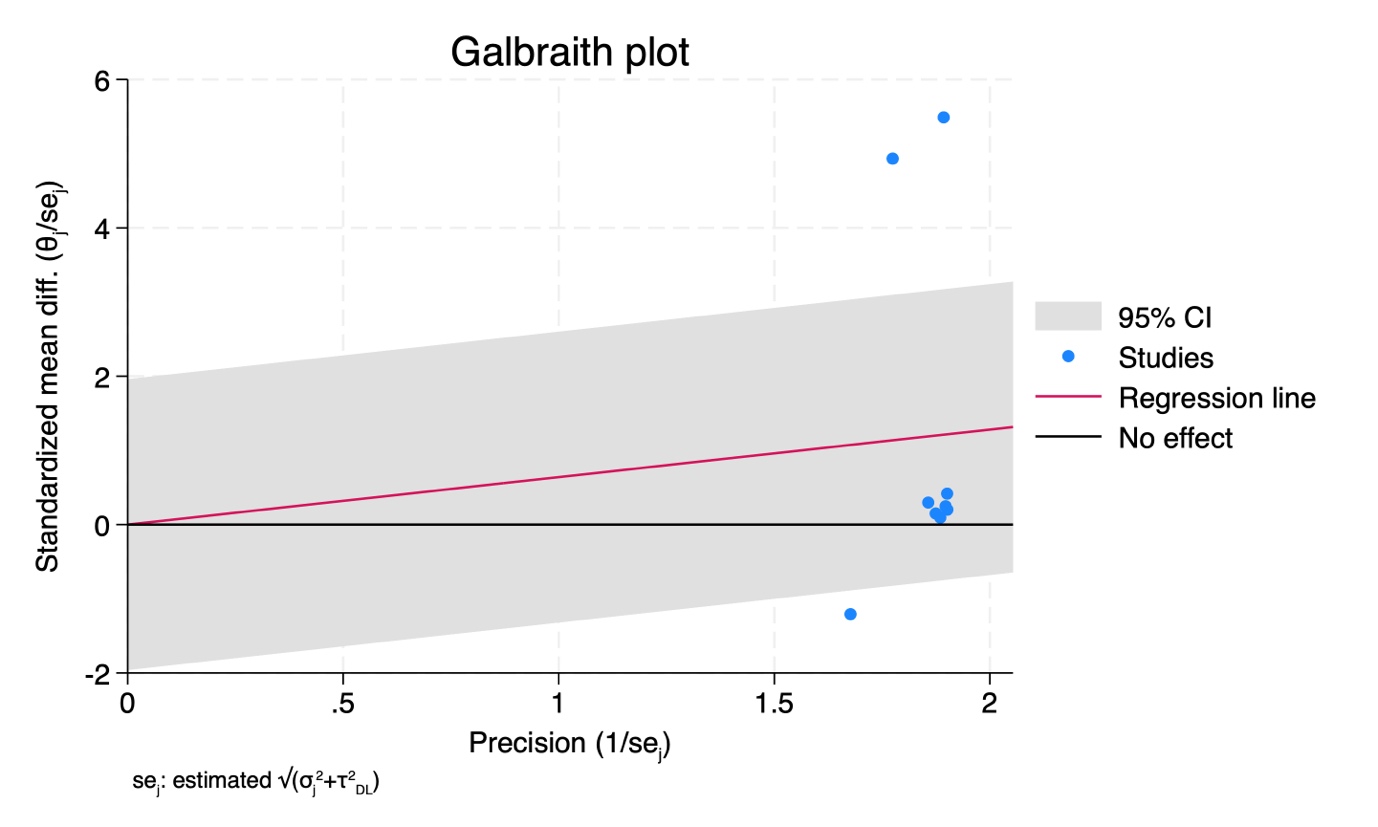


**Appendix 8 Peri-implant mucositis and peri-implantitis risk; other biological complications (including early failure) as reported by included studies.**

**S – smokers; NS – non-smokers; HS – heavy smokers**

| **Study** | **Peri-mucositis and peri-implantitis risk; Other biological complications (e.g soft tissue dehiscence, early implant failure)** |
| --- | --- |
| (Agliardi et al., 2023) | Failed implants due to peri-implantitis:  Maxilla: (total 288 implants)  n=3; at 107 months (S), 113 months (S), 102 months (S)  Mandible: (total 404 implants)  n=10; 4 at 73 months (S), 2 at 120 months (S), 4 at 60 months (NS)  Early failure: 1S, 2 in long-term use of corticosteroids, 1osteoporotic |
| (Alsahhaf et al., 2019) | No evidence of peri-implantitis or peri-implant mucositis at 3- and 5- year follow up |
| (Balaguer et al., 2015) | Failed implants due to peri-implantitis:  n=12 (total 360 implants)  average duration of functional loading: 52 months (includes 2 implants lost due to implant fracture) |
| (Bardis et al., 2023) | 24/178 implants with peri-implantitis:  S: 15/48  NS: 9/130  Risk factor (smoking) of peri-implantitis (univariate analysis):  OR/RR 6.111 95% CI 2.4566/15.207 B Coef. 0.846  Implants without biological complications (Kaplan-Meyer survival analysis:  15.420 SEM 0.387 %95 CI (14.662-16.178) |
| (Cha et al., 2014) | 5/462 implants removed ; 1 implant with acute infection & suppuration 1 month after surgery, 3 with loss of osseointegration during uncover surgery, 1 due to extensive peri-implant bone loss after 2 years of loading |
| (Chrcanovic et al., 2017) | 45/178 implants lost up to abutment connection or second stage; 85/178 implants lost up to 1-year post-surgery |
| (Deepa et al., 2018) | Failed implants due to peri-implantitis:  SSRI: n=6 / total implants 230 / total failures 25  non-SSRI: n=3 / total implants 450 / total failures 21 |
| (Degidi et al., 2016) | 1 patient (NS) reported moderate discomfort associated with redness and swelling in anterior maxilla 3 weeks post-surgery  1 patient reported prolonged sensorial disturbance after placement of 3-unit prosthesis (mandible near mental foramina area)  3 patients reported nuisance associated with moderate chewing difficulties in the days following surgery  Peri-implant mucositis: 35 implants (out of 193 available at 10-year follow up)  Peri-implantitis: 16 implants (out of 193 available at 10-year follow up) |
| (Doan et al., 2014) | 15/1241 implants had biologic complications requiring intervention; 5/15 peri-implantitis, 3/15 acute infection at surgical site during early healing period, 2/15 mucosal irritation caused by excess subgingival cement  19/1241 implants had aesthetic complications requiring intervention: 11/19 ‘kissing implant syndrome’; 12 implants had gingival recession (8/12 required gingival grafts) |
| (Garcia-Bellosta et al., 2010) | 24/980 early implant failure (before loading) |
| (He et al., 2015) | *Grouped based on bone density according to Lekholm and Zarb classification:*  G2 (total failures n=12)  3 (25%) early failures (lack of osseointegration), 5 (41.67%) occlusal overload, 1 (8.33%) peri-implantitis  G3 (total failures n=20)  9 (45%) early failures (lack of osseointegration), 6 (30%) occlusal overload, 2 (10%) peri-implantitis  G4 (total failures n=13)  6 (46.15%) early failures (lack of osseointegration), 4 (30.77%) occlusal overload, 2 (15.38%) peri-implantitis |
| (Hong et al., 2020) | 2/264 implants failed due to peri-implantitis (native bone group); 1 at 20 months after loading (NS) ; 1 at 18 months (S) |
| (Kandasamy et al., 2018) | S: 8/78 early failures, 2/78 late failures (unclear reason)  NS: 20/229 early failures, 4/229 late failures (unclear reason) |
| (Koldsland et al., 2009) | 9.2% subjects experienced early implant loss  6.4% subjects experienced late implant loss (these subjects also had at least one incidence of early implant loss) (unclear reason) |
| (Malo et al., 2015) | 16/1296 implants with radiolucency and mobility (were removed)  2/1296 implants had signs of persistent infection (were removed) |
| (Maló et al., 2018) | Implant failure:  1 failure at 43 months due to peri-implant pathology (S)  4 failures (2 at 15 months, 2 at 41 months) due to loss of implant integration (NS)  2 failures (at 6 months) due to loss of integration (S)  1 failure (at 33 months) due to progressive marginal bone loss, PD >6mm, suppuration (presented at 11 months) (S)  Biological complications: (11 NS and 13 S subject-level)  Implant infections; S: 4 implants / 4 patients; NS: 3 implants / 2 patients  Peri-implant pathology; S 11 implants / 9 patients; NS 7 implants / 9 patients |
| (Mangano et al., 2014) | 2/194 early failures due to lack of osseointegration (before prosthetic loading); 1 at 4 months (S), 1 at 3 months (NS) |
| (Peleg et al., 2006) | S:  Before loading: 10 due to infection/bone loss, 3 due to failure to integrate,  First year of loading: 3 due to infection  NS:  Before loading: 3 due to failure to integrate, 1 due to infection/bone loss  Late failure: 8 due to bone loss, 3 due to bone resorption  First year of loading: 13 due to infection |
| (Raes et al., 2015) | Midfacial recession:  S: 0.09±0.82 (median 0)  NS:0.52±0.89 (median 1)  Mesial papilla growth:  S:-0.21±1.31 (median -0.10)  NS:0.76±0.96 (median 1)  Distal papilla growth:  S: -0.13±1.52 (median 0)  NS: 0.63±1.02 (median 0.5) |
| (Tawil et al., 2008) | 6 early failures  Complications based on HbA1c:  <7%: 6/103 soft tissue complications, 0/103 peri-implantitis  7%-9%: 11/141 soft tissue complications, 6/141 peri-implantitis  >9%: 1/11 soft tissue complications, 1/11 peri-implantitis |
| (Vervaeke et al., 2012) | 9/1106 early failures (19 failures in total) |
| (Windael et al., 2020) | Distribution of implants with peri-implantitis:  Maxilla:  S: 34.4%  NS:9.8%  Mandible:  S: 22.2%  NS: 11.6% |

**Appendix 9 Quality of evidence based on GRADE**

| **Outcome** | **Number of studies** | **Design** | **Effect estimate** | **Quality of evidence** |
| --- | --- | --- | --- | --- |
| Implant failure (implant level) | 27 | Observational studies | OR = 0.32  [CI 95%: 0.20, 0.51] | Low  ⨁⨁◯◯^a^ |
| Implant failure (patient level) | 10 | Observational studies | OR = 0.43  [CI 95%: 0.20, 0.90] | Low  ⨁⨁◯◯^a^ |
| Crestal bone loss | 11 | Observational studies | 0.58 mm [-0.10, 1.25] | Low  ⨁⨁◯◯^a^ |

a: downgraded due to risk of bias and to inconsistency (heterogeinity). Observational studies

**References**

Abduljabbar, T., Al-Hamoudi, N., Al-Sowygh, Z. H., Alajmi, M., Javed, F. & Vohra, F. (2018) Comparison of peri-implant clinical and radiographic status around short (6 mm in length) dental implants placed in cigarette-smokers and never-smokers: Six-year follow-up results. *Clinical implant dentistry and related research* **20,** 21-25. doi:10.1111/cid.12564.

Agliardi, E. L., Pozzi, A., Romeo, D. & Del Fabbro, M. (2023) Clinical outcomes of full-arch immediate fixed prostheses supported by two axial and two tilted implants: A retrospective cohort study with 12-15 years of follow-up. *Clinical oral implants research* **34,** 351-366. doi:10.1111/clr.14047.

Al Amri, M. D., Kellesarian, S. V., Abduljabbar, T. S., Al Rifaiy, M. Q., Al Baker, A. M. & Al-Kheraif, A. A. (2017) Comparison of Peri-Implant Soft Tissue Parameters and Crestal Bone Loss Around Immediately Loaded and Delayed Loaded Implants in Smokers and Non-Smokers: 5-Year Follow-Up Results. *Journal of periodontology* **88,** 3-9. doi:10.1902/jop.2016.160427.

Alahmari, F., Javed, F., Ahmed, Z. U., Romanos, G. E. & Al-Kheraif, A. A. (2019) Soft tissue status and crestal bone loss around conventionally-loaded dental implants placed in cigarette- and waterpipe (narghile) smokers: 8-years' follow-up results. *Clinical implant dentistry and related research* **21,** 873-878. doi:10.1111/cid.12746.

Alazmi, S. O., Almutairi, F. J. & Alresheedi, B. A. (2021) Comparison of Peri-Implant Clinicoradiographic Parameters among Non-Smokers and Individuals Using Electronic Nicotine Delivery Systems at 8 Years of Follow-up. *Oral health & preventive dentistry* **19,** 511-516. doi:10.3290/j.ohpd.b2082123.

Alghamdi, O., Alrabiah, M., Al-Hamoudi, N., AlKindi, M., Vohra, F. & Abduljabbar, T. (2020) Peri-implant soft tissue status and crestal bone loss around immediately-loaded narrow-diameter implants placed in cigarette-smokers: 6-year follow-up results. *Clinical implant dentistry and related research* **22,** 220-225. doi:10.1111/cid.12893.

Alhenaki, A. M., Alrawi, F. K., Mohamed, A., Alshahrani, A., Alrabiah, M., Mokeem, S. A., AlHamdan, E. M., Ahmad, P., Vohra, F. & Abduljabbar, T. (2021) Clinical, radiographic and restorative parameters for short tuberosity implants placed in smokers: a retrospective study with 5 year follow-up. *Odontology* **109,** 979-986. doi:10.1007/s10266-021-00623-2.

Alsaadi, G., Quirynen, M., Komárek, A. & Van Steenberghe, D. (2008) Impact of local and systemic factors on the incidence of late oral implant loss. *Clinical oral implants research* **19,** 670-676. doi:10.1111/j.1600-0501.2008.01534.x.

Alsahhaf, A., Alshagroud, R. S., Al-Aali, K. A., Alofi, R. S., Vohra, F. & Abduljabbar, T. (2019) Survival of Titanium-Zirconium and Titanium Dental Implants in Cigarette-smokers and Never-smokers: A 5-Year Follow-up. *The Chinese journal of dental research : the official journal of the Scientific Section of the Chinese Stomatological Association (CSA)* **22,** 265-272. doi:10.3290/j.cjdr.a43737.

Balaguer, J., Ata-Ali, J., Peñarrocha-Oltra, D., García, B. & Peñarrocha-Diago, M. (2015) Long-term survival rates of implants supporting overdentures. *The Journal of oral implantology* **41,** 173-177. doi:10.1563/AAID-JOI-D-12-00178.

Bardis, D., Agop-Forna, D., Pelekanos, S., Chele, N., Dascălu, C., Török, R., Török, B., Cristea, I., Bardi, P. M. & Forna, N. (2023) Assessment of Various Risk Factors for Biological and Mechanical/Technical Complications in Fixed Implant Prosthetic Therapy: A Retrospective Study. *Diagnostics* **13**. doi:10.3390/diagnostics13142341.

Castellanos-Cosano, L., Carrasco-García, A., Corcuera-Flores, J. R., Silvestre-Rangil, J., Torres-Lagares, D. & Machuca-Portillo, G. (2021) An evaluation of peri-implant marginal bone loss according to implant type, surgical technique and prosthetic rehabilitation: a retrospective multicentre and cross-sectional cohort study. *Odontology* **109,** 649‐660. doi:10.1007/s10266-020-00587-9.

Cha, H.-S., Kim, A., Nowzari, H., Chang, H.-S. & Ahn, K.-M. (2014) Simultaneous sinus lift and implant installation: prospective study of consecutive two hundred seventeen sinus lift and four hundred sixty-two implants. *Clinical implant dentistry and related research* **16,** 337-347. doi:<https://dx.doi.org/10.1111/cid.12012>.

Chrcanovic, B. R., Kisch, J., Albrektsson, T. & Wennerberg, A. (2017) Intake of Proton Pump Inhibitors Is Associated with an Increased Risk of Dental Implant Failure. *The International journal of oral & maxillofacial implants* **32,** 1097-1102. doi:<https://dx.doi.org/10.11607/jomi.5662>.

Deepa, Mujawar, K., Dhillon, K., Jadhav, P., Das, I. & Singla, Y. K. (2018) Prognostic Implication of Selective Serotonin Reuptake Inhibitors in Osseointegration of Dental Implants: A 5-year Retrospective Study. *The journal of contemporary dental practice* **19,** 842-846. doi:10.5005/jp-journals-10024-2345.

Degidi, M., Nardi, D. & Piattelli, A. (2016) 10-year prospective cohort follow-up of immediately restored XiVE implants. *Clinical oral implants research* **27,** 694-700. doi:10.1111/clr.12642.

Doan, N. V., Du, Z., Reher, P. & Xiao, Y. (2014) Flapless dental implant surgery: a retrospective study of 1,241 consecutive implants. *The International journal of oral & maxillofacial implants* **29,** 650-658.

Garcia-Bellosta, S., Bravo, M., Subira, C. & Echeverria, J. J. (2010) Retrospective study of the long-term survival of 980 implants placed in a periodontal practice. *The International journal of oral & maxillofacial implants* **25,** 613-619.

He, J., Zhao, B., Deng, C., Shang, D. & Zhang, C. (2015) Assessment of implant cumulative survival rates in sites with different bone density and related prognostic factors: an 8-year retrospective study of 2,684 implants. *The International journal of oral & maxillofacial implants* **30,** 360-371. doi:10.11607/jomi.3580.

Hong, J. Y., Shin, E. Y., Herr, Y., Chung, J. H., Lim, H. C. & Shin, S. I. (2020) Implant survival and risk factor analysis in regenerated bone: results from a 5-year retrospective study. *Journal of periodontal & implant science* **50,** 379-391. doi:<https://dx.doi.org/10.5051/jpis.2002140107>.

Jesch, P., Jesch, W., Bruckmoser, E., Krebs, M., Kladek, T. & Seemann, R. (2018) An up to 17-year follow-up retrospective analysis of a minimally invasive, flapless approach: 18 945 implants in 7783 patients. *Clinical implant dentistry and related research* **20,** 393-402. doi:10.1111/cid.12593.

Kandasamy, B., Kaur, N., Tomar, G. K., Bharadwaj, A., Manual, L. & Chauhan, M. (2018) Long-term Retrospective Study based on Implant Success Rate in Patients with Risk Factor: 15-year Follow-up. *The journal of contemporary dental practice* **19,** 90-93.

Koldsland, O. C., Aamdal Scheie, A. & Merete Aass, A. (2009) Prevalence of implant loss and the influence of associated factors. *Journal of periodontology* **80,** 1069-1075. doi:10.1902/jop.2009.080594.

Malo, P., de Araujo Nobre, M., Lopes, A., Ferro, A. & Gravito, I. (2015) All-on-4 R Treatment Concept for the Rehabilitation of the Completely Edentulous Mandible: A 7-Year Clinical and 5-Year Radiographic Retrospective Case Series with Risk Assessment for Implant Failure and Marginal Bone Level. *Clinical implant dentistry and related research* **17 Suppl 2,** e531-541. doi:<https://dx.doi.org/10.1111/cid.12282>.

Maló, P. S., de Araújo Nobre, M. A., Ferro, A. S. & Parreira, G. G. (2018) Five-year outcome of a retrospective cohort study comparing smokers vs. nonsmokers with full-arch mandibular implant-supported rehabilitation using the All-on-4 concept. *Journal of oral science* **60,** 177-186. doi:10.2334/josnusd.16-0890.

Mangano, F. G., Shibli, J. A., Sammons, R. L., Iaculli, F., Piattelli, A. & Mangano, C. (2014) Short (8-mm) locking-taper implants supporting single crowns in posterior region: a prospective clinical study with 1-to 10-years of follow-up. *Clinical oral implants research* **25,** 933-940. doi:10.1111/clr.12181.

Mundt, T., Mack, F., Schwahn, C. & Biffar, R. (2006) Private practice results of screw-type tapered implants: Survival and evaluation of risk factors. *International Journal of Oral and Maxillofacial Implants* **21,** 607-614.

Nitzan, D., Mamlider, A., Levin, L. & Schwartz-Arad, D. (2005) Impact of smoking on marginal bone loss. *The International journal of oral & maxillofacial implants* **20,** 605-609.

Peleg, M., Garg, A. K. & Mazor, Z. (2006) Healing in smokers versus nonsmokers: Survival rates for sinus floor augmentation with simultaneous implant placement. *International Journal of Oral and Maxillofacial Implants* **21,** 551-559.

Raes, S., Rocci, A., Raes, F., Cooper, L., De Bruyn, H. & Cosyn, J. (2015) A prospective cohort study on the impact of smoking on soft tissue alterations around single implants. *Clinical oral implants research* **26,** 1086-1090. doi:10.1111/clr.12405.

Sánchez-Pérez, A., Moya-Villaescusa, M. J. & Caffesse, R. G. (2007) Tobacco as a risk factor for survival of dental implants. *Journal of periodontology* **78,** 351-359. doi:10.1902/jop.2007.060299.

Sun, C., Zhao, J., Jianghao, C. & Hong, T. (2016) Effect of Heavy Smoking on Dental Implants Placed in Male Patients Posterior Mandibles: A Prospective Clinical Study. *The Journal of oral implantology* **42,** 477-483. doi:10.1563/aaid-joi-D-16-00078.

Tawil, G., Younan, R., Azar, P. & Sleilati, G. (2008) Conventional and advanced implant treatment in the type II diabetic patient: surgical protocol and long-term clinical results. *The International journal of oral & maxillofacial implants* **23,** 744-752.

Vervaeke, S., Collaert, B., Vandeweghe, S., Cosyn, J., Deschepper, E. & De Bruyn, H. (2012) The effect of smoking on survival and bone loss of implants with a fluoride-modified surface: a 2-year retrospective analysis of 1106 implants placed in daily practice. *Clinical Oral Implants Research* **23,** 758-766. doi:10.1111/j.1600-0501.2011.02201.x.

Wach, T., Hadrowicz, P., Trybek, G., Michcik, A. & Kozakiewicz, M. (2023) Is Corticalization in Radiographs Related to a Higher Risk of Bone Loss around Dental Implants in Smoking Patients? A 5-Year Observation of Radiograph Bone-Texture Changes. *Journal of clinical medicine* **12**. doi:10.3390/jcm12165351.

Windael, S., Vervaeke, S., De Buyser, S., De Bruyn, H. & Collaert, B. (2020) The long-term effect of smoking on 10 years’ survival and success of dental implants: A prospective analysis of 453 implants in a non-university setting. *Journal of clinical medicine* **9**. doi:10.3390/jcm9041056.

Zhang, Y., Li, S., Di, P., Zhang, Y., Wu, A. & Lin, Y. (2023) Comparison of 4- or 6-implant supported immediate full-arch fixed prostheses: A retrospective cohort study of 217 patients followed up for 3-13 years. *Clinical implant dentistry and related research* **25,** 381-397. doi:10.1111/cid.13170.

Zuffetti, F., Testarelli, L., Bertani, P., Vassilopoulos, S., Testori, T. & Guarnieri, R. (2020) A Retrospective Multicenter Study on Short Implants With a Laser-Microgrooved Collar (≤7.5 mm) in Posterior Edentulous Areas: Radiographic and Clinical Results up to 3 to 5 Years. *Journal of oral and maxillofacial surgery* **78,** 217-227. doi:10.1016/j.joms.2019.08.007.
